# Insights into molecular recognition from the crystal structures of *p-tert*-butyl­calix[6]arene complexed with different solvents

**DOI:** 10.1107/S2052252521010678

**Published:** 2021-11-16

**Authors:** Maura Malinska

**Affiliations:** a Faculty of Chemistry, University of Warsaw, Pasteura 1, Warsaw, Poland

**Keywords:** calixarenes, molecular recognition, intermolecular interactions, hydrogen bonding

## Abstract

Calixarenes are host molecules that can form complexes with one or more guest molecules, and molecular recognition in calixarenes can be affected by many factors. With a view to establishing molecular recognition rules, the host *p-tert*-butyl­calix[6]arene (TBC6) was crystallized with different guest molecules (cyclo­hexane, anisole, heptane, toluene, benzene, methyl acetate, ethyl acetate, di­chloro­methane, tetra­hydro­furan and pyridine) and the obtained structures were characterized by X-ray diffraction. With most solvents, 1:1 and/or 1:3 host–guest complexes were formed, although other stoichiometries were also observed with small guest molecules, and crystallization from ethyl acetate produced the unsolvated form. The calculated fill percentage of the TBC6 cavity was ∼55% for apolar guests and significantly lower for polar solvents, indicating that polar molecules can bind to apolar cavities with significantly lower packing coefficients. The most stable crystals were formed by 1:1 host–guest inclusion complexes. The ratio between the apolar surface area and the volume was used to predict the formation of inclusion versus exclusion complexes, with inclusion complexes observed at ratios <40. These findings allow the binding of potential guest molecules to be predicted and a suitable crystal packing for the designed properties to be obtained.

## Abstract 2

With a view to establishing molecular recognition rules, the host *p*-*tert*-butyl­calix[6]arene was crystallized with different guest molecules. The ratio between the apolar surface area and the volume was used to predict the formation of inclusion versus exclusion complexes, with inclusion complexes observed at ratios <40.

## 1. Introduction

Calixarenes, which are vase-like molecules, are widely used in supramolecular chemistry due to the fact that they can be prepared relatively easily, and can be selectively functional­ized at different positions to obtain desired shapes and functions (Gutsche, 2008 ▸). Calixarenes have been applied in various fields, including in biology (Danylyuk & Suwinska, 2009 ▸; Danylyuk & Fedin, 2012 ▸) and in the design of pharmaceutical agents (Yousaf *et al.*, 2015 ▸; Da Silva *et al.*, 2004 ▸; Nimse & Kim, 2013 ▸). They have also been shown to form metal nanoparticles (Zhou *et al.*, 2014 ▸; Pulkkinen *et al.*, 2014 ▸) and organic nanoparticles (Jiang *et al.*, 2014 ▸), in addition to serving as the basis for sensor construction (Montmeat *et al.*, 2014 ▸) and being applicable as extractants for the *f*-block elements.

The calix[*n*]arene molecular skeleton consists of a series of phenol rings linked by methyl­ene groups, and structural variants can be obtained by changing the bridging unit or the number of phenolic hydroxyl functionalities. Owing to their flexible and geometrically variable interior cavity, which is large enough to accommodate one or more smaller molecules, calix[*n*]arenes can function as molecular containers or host molecules (Liao *et al.*, 2009 ▸; Dalgarno *et al.*, 2006 ▸; Leśniewska *et al.*, 2019 ▸). The formation of their host–guest complexes is stabilized by intermolecular forces, such as ionic interactions, hydrogen bonding, π–π interactions, hydro­phobic forces and van der Waals forces.

Cornforth *et al.* (1955 ▸, 1973 ▸) first recognized the ability of calix[4]arenes to assume four conformations, in which various numbers of aryl groups project upward or downward relative to an average plane defined by the bridging methyl­ene groups. The calix[5]arenes also have only four true ‘up/down’ conformers, whereas the calix[6]arenes have eight and the calix[8]arenes have sixteen. To date, most research in this field has been devoted to the calix[4]arenes, since they can be modified selectively and are easier to crystallize. The cavity size of the basic calix[4]arene is small, and so in the context of host–guest chemistry, the application of the larger calix[6]arenes could be advantageous; however, the structures and properties of larger calixarenes have received little research attention.

The conformations of the parent calix[6]arene have been described as distorted-cone, pinched-cone and 1,2,3-alternate structures. In this context, Bott and co-workers (Wolfgong *et al.*, 1996 ▸) showed that the conformation in the solid state is a function of the solvent from which the compound is crystallized. When the solvent (*e.g.* benzene) cannot hydrogen bond with the OH groups of the calixarene, the pinched-cone conformation is obtained, in which all the OH groups are intramolecularly hydrogen bonded in a cyclic array. However, when the solvent [*e.g.* acetone or di­methyl sulfoxide (DMSO)] can disrupt the intramolecular hydrogen bonding, the calixarene assumes a distorted 1,2,3-alternate conformation. However, pyridine (Malinska, 2021 ▸) and DMSO (Martins *et al.*, 2017 ▸), which are both molecules that can form hydrogen bonds, crystallize with *p-tert*-butyl­calix[6]arene (TBC6) in a winged-cone conformation. This conformation exists only in the metastable crystal structure at room temperature, and dissolves to form crystal structures with lower host–guest ratios or pinched-cone conformations. TBC6 without solvent molecules has only been obtained by sublimation (Galindo-García & Torres, 2020 ▸).

A study by Mecozzi & Rebek (1998 ▸) based on several supramolecular hydrogen-bonded capsules with internal cavities of different sizes led to the proposal of the 55% rule, which states that the hydro­phobic space of a host is energetically best filled with a lipophilic shape-complementary guest at a packing coefficient of 0.55 ± 0.09. Studies on guest complexation in the highly confined spaces of supramolecular capsules and container molecules have confirmed that shape-complementary guests have an optimal size (Scarso *et al.*, 2003 ▸; Trembleau & Rebek, 2003 ▸; Zhang *et al.*, 2014 ▸; Gottschalk *et al.*, 2008 ▸). For lipophilic guests, the optimal packing coefficient is approximately 55%, but this value increases to approximately 63% when additional polar interactions are established (Tsuzuki *et al.*, 2006 ▸; Shibasaki *et al.*, 2007 ▸; Nishio *et al.*, 2014 ▸; Hornung *et al.*, 2011 ▸). However, in protein–ligand systems, which also possess a dominating hydro­phobic character, the greatest correlation has been found between the binding free energy and the burial of apolar surfaces upon complex formation (Olsson *et al.*, 2008 ▸). Therefore, to understand molecular recognition in calixarenes, many potential factors should be considered, including the sizes and shapes of the guest molecules and the host binding cavity, the polarity, and the propensity to form hydrogen bonds and other noncovalent interactions.

Thus, in this study, the crystallization of TBC6 with different solvents is investigated, and the crystal structures of the obtained complexes are determined to establish general molecular recognition rules. The focus of this study is to establish guest-molecule properties based on new complexes and also on complexes found in the Cambridge Structural Database (CSD) (Groom *et al.*, 2016 ▸) that enable the prediction of potential new guests for binding to the calixarene cavities to yield designed crystal structures. Additionally, crystal structure analysis is completed by carrying out interaction energy calculations to understand the general trends in host–guest complex formation that centre on the energy rather than the geometric parameters and interaction types.

## 2. Experimental

### 2.1. Crystallization

Crystals of inclusion complexes were obtained by crystallization from saturated solutions of the host (TBC6) with various guests, *i.e.* cyclo­hexane, anisole, *n*-heptane, toluene, benzene, methyl acetate, ethyl acetate, di­chloro­methane (DCM), tetra­hydro­furan (THF) and pyridine. Saturated solutions were prepared by dissolving the host in warm solvent under gentle stirring. The solutions were then slowly cooled and the solvent allowed to evaporate, which resulted in crystallization of the desired inclusion compounds. TBC6 crystallized from benzene to give structure **1**; from pyridine to give structures **2** and **8**; from DCM to give structures **3**, **12** and **13**; from cyclo­hexane to give structure **4**; from toluene to give structure **5**; from methyl acetate to give structure **6**; from THF to give structure **7**; from anisole to give structure **9**; from *n*-heptane to give structure **10**; and from ethyl acetate to give structure **11**. Crystal **3**, which possesses a host–guest ratio of 1:3, was obtained from supersaturated DCM solutions. However, when TBC6 was crystallized from an undersaturated DCM solution at 303.15 K, more complexes with 1:4 (**12**) and 1:2 (**13**) host–guest ratios were observed. In addition, we found that structure **12** grew in more saturated solutions than structure **13**. Specific details are found in the supporting information (Table S1).

### 2.2. X-ray diffraction

Single-crystal X-ray diffraction (XRD) measurements of structures **1**–**13** were carried out on an Agilent Technologies Xcalibur CCD diffractometer equipped with a copper or molybdenum sealed X-ray tube (Mo *K* radiation, λ = 0.71073 Å; Cu *K*, λ = 1.54184 Å), a graphite monochromator and a nitro­gen gas-flow device (Oxford Cryosystems). In all cases, single crystals of suitable sizes were mounted on a goniometer head using Paratone N oil and cooled to 100 K. Data collection strategies, based solely on ω scans, were optimized by applying the appropriate algorithms implemented within *CrysAlisPro* (Rigaku Oxford Diffraction, 2020 ▸). Unit-cell parameter determination, raw diffraction image integration, Lorentz and polarization corrections, oblique incidence effects, multiscan absorption corrections and frame-to-frame scaling were performed using the diffractometer software. The final data collection parameters are summarized in Tables S2–S4 in the supporting information. Crystal structures **2** and **8** were previously reported (Malinska, 2021 ▸) and cif files were deposited in the CSD as ELULOV01 and ELUMAC.

### 2.3. Structure solution and refinement

All structures were solved using direct methods implemented in the *SHELXT* program (Sheldrick, 2015*b* ▸) and refined with the *SHELXL* program (Sheldrick, 2015*a* ▸) within the independent atom model (IAM) approximation. In all cases, the positions of the hydrogen atoms were constrained. Positional disorder was observed in all the crystal structures, and some restraints and constraints were applied during structure refinement to achieve reasonable geometric parameters and anisotropic displacement parameters (ADPs). Details are available in the supporting information.

### 2.4. Crystal structures of TBC6 in the CSD

We analysed the CSD (Version 5.42, November 2020 plus two updates) to find crystal structures of TBC6 in pinched-cone conformations. The CSD was queried for error-free organic crystal structures determined at room temperature or below. A total of ten different crystal structures were found: KAHJUY (benzene 1:3; Halit *et al.*, 1988 ▸), KENBUA (tetra­chloro­ethene 1:1; Andreetti *et al.*, 1989 ▸), LODNIB (carbon di­sulfide 1:1; Schatz, Schildbach *et al.*, 2000 ▸), TECDUB (DCM 1:2; Felsmann *et al.*, 2006 ▸), UWIVUS (chloro­benzene 1:1; Ramon *et al.*, 2011 ▸), UWIWAZ (bromo­benzene 1:1; Ramon *et al.*, 2011 ▸), VARGOL (aceto­nitrile 2:3; Dale *et al.*, 2003 ▸), VARGUR (aceto­nitrile 1:2; Dale *et al.*, 2003 ▸), WORMEV (toluene 1:1; Lu *et al.*, 1999 ▸) and ZIPYOM (aceto­nitrile 2:3; Thuéry *et al.*, 1994 ▸).

### 2.5. Voids and binding-pocket volume

The voids function in *Mercury* (Macrae *et al.*, 2020 ▸) allows any empty spaces (voids) in a crystal structure that are big enough to contain a spherical ‘probe’ of a given radius to be found. After removing the solvent molecules, a probe with a radius of 1.2 Å was used to calculate the voids using the solvent-accessible surface. This surface and the ways in which it can be found are described elsewhere (Barbour, 2006 ▸). The voids were calculated for each crystal structure after removing the solvent molecules to estimate the volume of the TBC6 cavity. The surface area and volume of the solvent molecules were calculated using *Vega ZZ* software (Pedretti *et al.*, 2004 ▸).

### 2.6. Hirshfeld surface analysis

Structural frameworks (Turner *et al.*, 2015 ▸) for structures **1**–**13** were generated using the *CrystalExplorer* program (Turner *et al.*, 2017 ▸) (Figs. S1–S9). The energy framework facilitates the calculation of the interaction energies between all molecules in a cluster. The *CrystalExplorer* method is based on the *PIXEL* method (Gavezzotti, 2008 ▸), which is a semi-empirical technique for evaluating intermolecular interactions, and is based on integrating the calculated electron densities of single molecules. The monomer wavefunction was used to obtain accurate values for the electrostatic, polarization and repulsion energies using Grimme’s D2 dispersion correction (Grimme, 2011 ▸; Grimme *et al.*, 2011 ▸) and scaled appropriately (Mackenzie *et al.*, 2017 ▸). Hydrogen bonds were extended to the mean neutron values using the *LSDB* program (Volkov *et al.*, 2007 ▸). Owing to the presence of disordered regions in these structures, calculations were only performed for regions with the highest occupancy factors for the structures determined by us (**1**–**13**) and for those from the CSD (KENBUA, LODNIB, UWIVUS, UWIWAZ, VARGOL and VARGUR) after the addition of missing hydrogen atoms with the aid of *Mercury* (Macrae *et al.*, 2020 ▸). Hartree–Fock calculations with the 3-21G basis set were used. The results for all frameworks are presented using a scaling factor of 150 and an energy threshold of −15 kJ mol^−1^. Details can be found in the supporting information.

### 2.7. Theoretical calculations

Intermolecular interaction energies were evaluated using the *GAUSSIAN16* package (Frisch *et al.*, 2016 ▸). The density functional theory (DFT) (B3LYP)/6-31G** method was employed with the Grimme empirical dispersion correction (Grimme *et al.*, 2010 ▸) modified by the Becke–Johnson damping function (Grimme *et al.*, 2011 ▸, 2010 ▸) and correction for basis set superposition error (BSSE) (Boys & Bernardi, 1970 ▸; Simon *et al.*, 1996 ▸).

## 3. Results and discussion

### 3.1. Crystal structures

**Figure 1:**
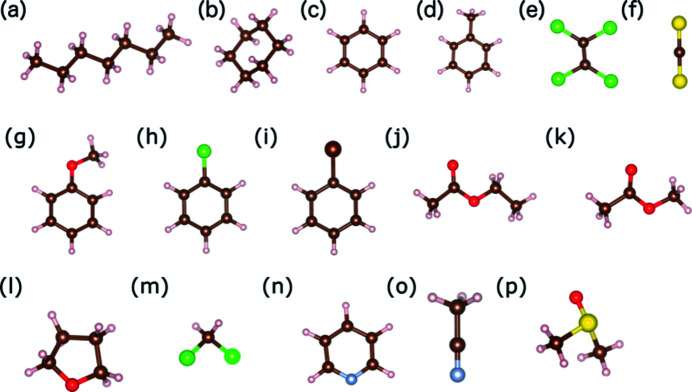
The guest molecules crystallized with TBC6 in this work, arranged in order of increasing polarity: (*a*) *n*-heptane, (*b*) cyclo­hexane, (*c*) benzene, (*d*) toluene, (*e*) tetra­chloro­ethyl­ene, (*f*) carbon di­sulfide, (*g*) anisole, (*h*) chloro­benzene, (*i*) bromo­benzene, (*j*) ethyl acetate, (*k*) methyl acetate, (*l*) tetrahydrofuran (THF), (*m*) dichloromethane (DCM), (*n*) pyridine and (*o*) dimethyl sulfoxide (DMSO).

One feature of calixarenes is their tendency to crystallize as solvates (Andreetti *et al.*, 1989 ▸; Ramon *et al.*, 2011 ▸). Among the compounds investigated in this study (Fig. 1 ▸), a non-solvated form was only obtained when TBC6 was crystallized from ethyl acetate (**11**), whereas the remaining solvents formed solvates with 1:3 stoichiometries [benzene (**1**), pyridine (**2**) and DCM (**3**)] or 1:1 stoichiometries [cyclo­hexane (**4**), toluene (**5**), methyl acetate (**6**), THF (**7**), pyridine (**8**), anisole (**9**) and *n*-heptane (**10**)]. DCM also formed complexes with other stoichiometries, *i.e.* 1:2 (**12**) and 1:4 (**13**). In most cases, well-shaped colourless single crystals were obtained by slow evaporation of the solutions at 298 K. It is worth noting that both pyridine solvate forms (1:3 and 1:1) grew simultaneously from the solution, as detailed elsewhere (Malinska, 2021 ▸). Crystallization from other common organic solvents (ethanol, propan-1-ol, propan-2-ol, acetone and chloro­form) led to powdered solids rather than any other solvated crystal structures.

**Figure 2:**
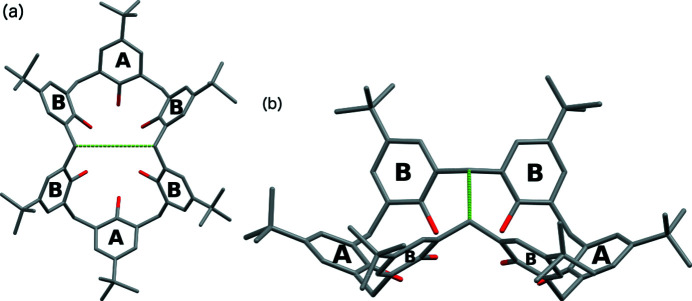
(*a*) Top and (*b*) side views of the TBC6 molecular structure in the pinched-cone conformation, showing the *A*- and *B*-type phenyl rings and definition of the *S* distance (green lines). The hydrogen atoms have been removed for clarity.

All compounds investigated in this study crystallized in centrosymmetric space groups, 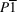 or *P*2_1_/*n*, with one TBC6 molecule in the asymmetric part of the unit cell. Here, only the structures with the pinched-cone conformation are discussed. The crystal structures with winged-cone (Martins *et al.*, 2017 ▸) and 1,2,3-alternate conformations (Wolfgong *et al.*, 1996 ▸) have been reported previously. To describe clearly the interactions of the solvent molecules with the calixarene structure, the phenyl rings of the calixarene were labelled as *A*- and *B*-type rings (Fig. 2 ▸).

### 3.2. 1:3 Host–guest structures

**Figure 3:**
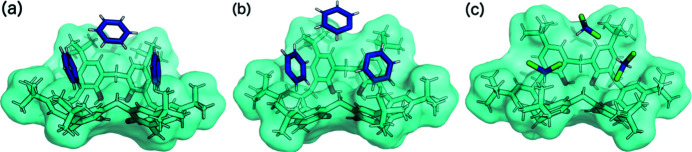
The asymmetric units of TBC6 solvated with (*a*) benzene (**1**), (*b*) pyridine (1:3, **2**) and (*c*) DCM (1:3, **3**). Disorder has been removed for clarity. From right to left, the guest molecules in the binding pockets represent dimer motifs I, I′′ and I′.

As shown by the example of pyridine complexes with TBC6, the crystal structures tend to transform into their less solvated structures, which are the thermodynamically more stable forms (Wolfgong *et al.*, 1996 ▸). As shown in Fig. 3 ▸, host–guest crystal structures with 1:3 ratios were formed from benzene (**1**), pyridine (**2**) and DCM (**3**). Two of the three molecules occupy the binding cavities of the macrocycle, whereas the third is situated above it, between two *tert*-butyl (^*t*^Bu) groups (Fig. 3 ▸).

**Figure 4:**
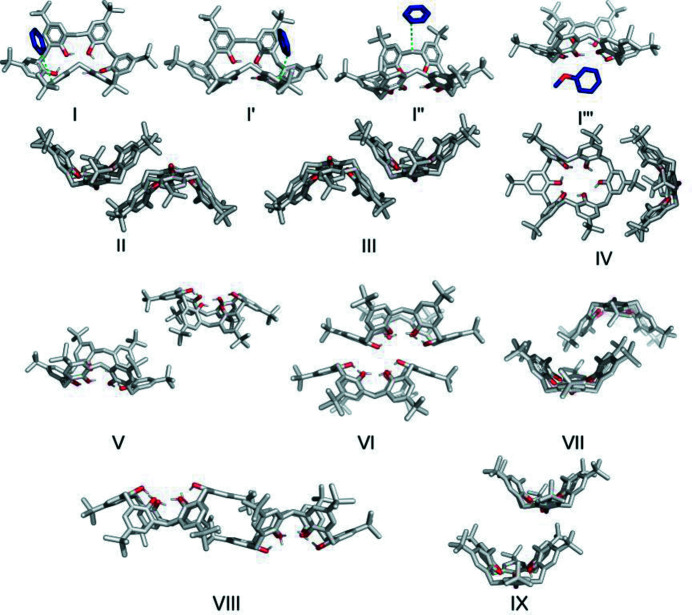
Selected dimer motifs extracted from the guest–TBC6 crystal structures.

In structure **1**, one of the benzene molecules forms three contacts with the macrocycle [motif I, Fig. 4 ▸(*a*)], including a C—H⋯π stacking interaction [distance between atoms C72 and C25 = 3.752 (3) Å]. In addition, this molecule is locked from the top by two ^*t*^Bu groups with distances of 3.454 (3) between atoms C69 and C54(−*x*, 1 − *y*, 1 − *z*) and 3.696 (3) Å between atoms C70 and C64(−*x*, 1 − *y*, 1 − *z*). Furthermore, a C—H⋯O contact is formed with a distance between atoms C70 and O1(−1 + *x*, *y*, *z*) of 3.531 (3) Å. The benzene mol­ecule of motif I′ in structure **1** is disordered between two positions and a C—H⋯π interaction is also formed in this case [Fig. 4 ▸(*b*)]. However, from the top, the benzene molecule is only surrounded by the ^*t*^Bu groups, which can also rotate. The third benzene molecule (motif I′′) acts as an acceptor in a C—H⋯π interaction with the methyl­ene bridge as a donor. The contact distance between atoms C22 and C78 is 3.746 (3) Å [Fig. 4 ▸(*c*)]. Crystal **2** is isostructural, with three pyridine molecules interacting through C—H⋯π contacts with TBC6.

In structure **3**, the DCM molecules occupy the same binding pocket in TBC6. The first molecule (motif I) exhibits a C—H⋯π interaction with a distance of 3.67 (1) Å between atoms C67 and C61. The second molecule forms the same type of interaction [distance between atoms C68 and C48 = 3.664 (8) Å] and a Cl⋯π contact [distance between atoms Cl3 and C23 = 3.310 (6) Å]. Motif I′′ corresponds to the trapping of DCM molecules between two TBC6 molecules, wherein a lack of directional contact results in disorder over two positions.

**Figure 5:**
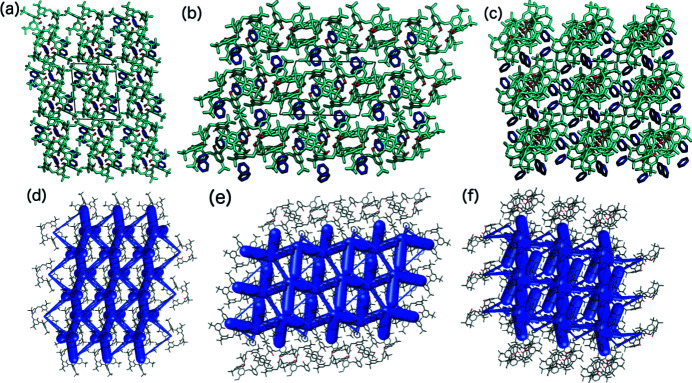
(*a*)–(*c*) Packing diagrams and (*d*)–(*f*) crystal structure frameworks of **1**, viewed along the [100], [001] and [110] axes from left to right, respectively.

Along the [100] axes in structures **1** and **2**, the macrocycles and solvent molecules form a column with the cavities in the same direction (Fig. 5 ▸), and the next column is antiparallel with the cavities in the opposite direction. Along the [001] direction, each column is surrounded by solvent molecules. In contrast, along the [110] direction, an off-set bilayer packing motif exists, and the same motif is present in structure **3**. However, as DCM molecules have a smaller volume, the space required by the solvent is smaller, and so instead of the columns packing side by side, as in structures **1** and **2**, the columns are shifted by half the macrocycle diameter, resulting in denser packing and a change in the space group from triclinic 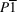 to monoclinic *P*2_1_/*n*.

Analysis of the energy of interactions for a selected dimer shows that the guest molecules (benzene, pyridine and DCM) form motif I with similar interactions from an energetic point of view (approximately −50 kJ mol^−1^, Table 1 ▸), wherein the strength of these interactions can be attributed to dispersion interactions. The interaction energy of motif I was validated by counterpoise calculations, which confirmed this trend (Table S24). The second (I′) and third (I′′) interactions have similar or higher total interaction energies. The TBC6 molecules form an off-set bilayer through two structural motifs, which are similar (motifs II and III, Fig. 4 ▸). Both involve stacking and C—H⋯π interactions, but more importantly, shape complementarity can be achieved owing to rotation of the molecule. Although the predominant contribution to the total energy is the dispersion energy, electrostatics also play a role because of this rotation and the antiparallel orientation of the molecular dipole moment. In contrast, structure **3** exhibits two identical motifs II with an interaction energy of −124.4 kJ mol^−1^.

### 3.3. 1:1 Host–guest structures with small guests

**Figure 6:**
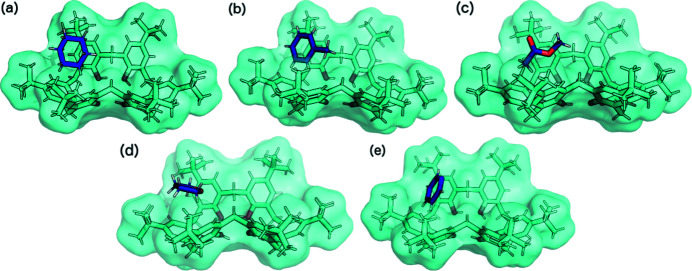
The asymmetric units of TBC6 solvated with (*a*) cyclo­hexane (**4**), (*b*) toluene (**5**), (*c*) methyl acetate (**6**), (*d*) THF (**7**) and (*e*) pyridine (1:1, **8**). Disorder has been removed for clarity.

**Figure 7:**
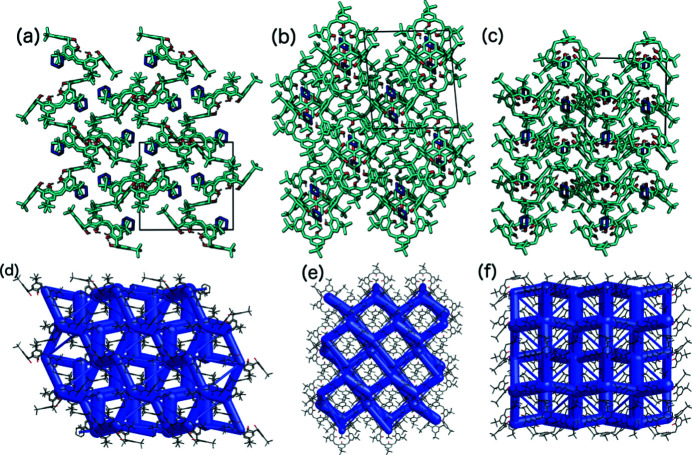
(*a*)–(*c*) The crystal packing and (*d*)–(*f*) the crystal framework of **4**: (*a*), (*d*) viewed along the [100] axis, (*b*), (*e*) viewed along the [010] axis, and (*c*), (*f*) viewed along the [001] axis. Note that this structure is isostructural with structures **5**–**8**.

As previously shown, the crystallization of TBC6 with pyridine undergoes a crystal transformation from a higher host–guest ratio (Malinska, 2021 ▸). Crystals with 1:1 ratios are more stable, and were formed by most of the examined systems. Structures containing cyclo­hexane (**4**), toluene (**5**), methyl acetate (**6**), THF (**7**), pyridine (1:1, **8**), tetra­chloro­ethyl­ene (KENBUA), carbon disulfide (LODNIB), chloro­benzene (UWIVUS) and bromo­benzene (UWIWAZ), which crystallized in the *P*2_1_/*n* space group with *Z* = 4, all formed isostructural crystals. In these structures, the host molecule is in the pinched-cone conformation stabilized by intramolecular hydrogen bonds on the lower rim, while the guest molecule resides in one of the two cavities. Many of the ^*t*^Bu groups have two orientations. The smallest guest molecules (methyl acetate and THF) and benzene derivatives are disordered over two positions, whereas the other guests have one conformation. The site occupancies for the two positions of methyl acetate and THF [Figs. 6 ▸(*c*) and 6 ▸(*d*)] were refined to 0.86 and 0.64, respectively, for the major disordered part. As shown by the packing diagram (Fig. 7 ▸), these structures consist of layers that intercalate along the [010] axis. In contrast, the guest mol­ecules and ^*t*^Bu groups are observed to be in ‘cavities’ along the [100] axis [Fig. 7 ▸(*a*)].

All guests, with the exception of methyl acetate, tetra­chloro­ethyl­ene and carbon di­sulfide, are stabilized by C—H⋯π interactions in the TBC6 cavity (Table 2 ▸). Moreover, the interaction energies are similar (approximately −50 kJ mol^−1^) for all the guests in these structures (motif I, Table 1 ▸). The second cavity of TBC6 is not occupied by solvent molecules; instead, a ^*t*^Bu group from an *A*-type phenyl group fills this space (motif IV, Fig. 4 ▸), with an interaction strength of approximately −80 kJ mol^−1^ (Table 1 ▸). This motif forms a 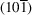 criss-cross framework, as observed along the [010] axis [Figs. 7 ▸(*b*) and 7 ▸(*e*)]. Along the [001] axis, motifs II and III result in an off-set bilayer chain, and these interactions are the strongest in this crystal structure type [Fig. 7 ▸(*f*)]. These interactions possess a contribution from electrostatic forces but are still dominated by dispersion interactions.

### 3.4. 1:1 Host–guest structures with large guests

**Figure 8:**
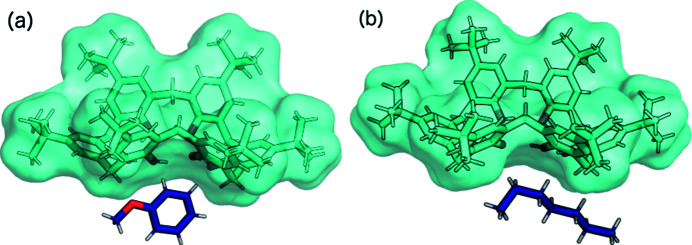
The asymmetric units of TBC6 solvated with (*a*) anisole (**9**) and (*b*) *n*-heptane (**10**). Disorder has been removed for clarity.

Anisole and *n*-heptane are too large to fit into the TBC6 cavities. Therefore, they are located outside the TBC6 next to a methyl­ene bridge pointing towards the inside of the macrocycle (Fig. 8 ▸). The interaction energy for motif I′′′ (Fig. 4 ▸) is weaker than that for motif I (−33.6 and −39.5 kJ mol^−1^ for anisole and *n*-heptane, respectively). The placement of the guest prevents the formation of the strongest structural motifs (II and III); instead, TBC6 interacts via its hydro­philic rim (motif VI, Fig. 4 ▸). However, there it still has a major contribution from dispersion (−161.8 kJ mol^−1^), whereas the electro­static interactions are repulsive (7.8 kJ mol^−1^). The closest contact is between symmetry-related oxygen atoms O1 and O1(1 − *x*, 1 − *y*, 1 − *z*) with a distance of 2.863 (7) Å in structure **9**. An analogous contact between atoms O4 and O4(−*x*, 1 − *y*, 2 − *z*) with a distance of 2.613 (3) Å was observed for structure **10**.

**Figure 9:**
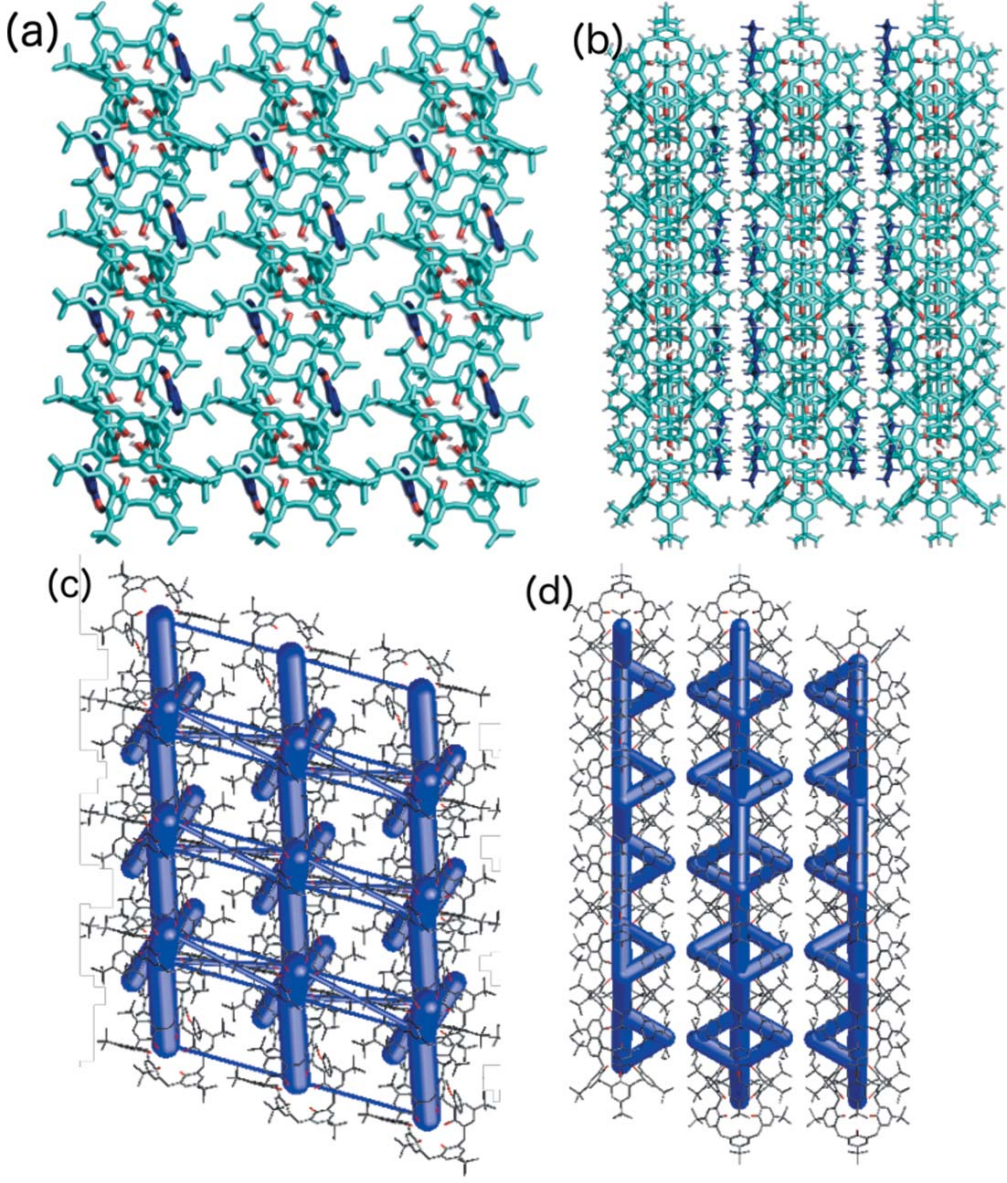
(*a*), (*b*) The crystal packing and (*c*), (*d*) the crystal frameworks of **9** and **10** viewed along the [001] axis. Disorder has been removed for clarity.

In these structures, both cavities in the pinched-cone conformation are occupied by ^*t*^Bu groups. However, the TBC6 molecules form different motifs in these structures. A new structural motif (VII) is observed in structure **9**, in which the cavity contains the ^*t*^Bu group of the *B*-type phenyl ring that is more perpendicular to the average plane, as determined by the oxygen atoms (Fig. 4 ▸). In contrast, in structure **10**, motif IV is observed, as in the case of structures **4**–**8**. The interaction energies in this case are −84.7 and −68.9 kJ mol^−1^, since there are two similar motifs in the structure. Both structures form columns along the [001] axis through motif VI (Fig. 9 ▸). From the top and bottom, four ^*t*^Bu groups fill the cavities through motifs VII and IV in structures **9** and **10**, respectively. The guest molecules are situated on both sides of the columns. In this case, the interaction between columns is significantly weaker (motif V), with interaction energies of −27.1 and −10.5 kJ mol^−1^ being determined for structures **9** and **10**, respectively. Furthermore, the distance between the columns in the crystal with *n*-heptane (**10**) is significantly longer.

### 3.5. Other host–guest structures

**Figure 10:**
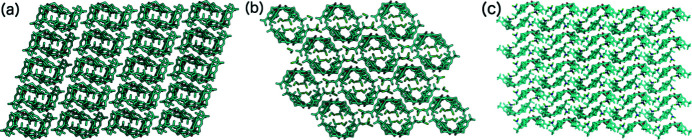
The crystal packing of **11**, **12** and **13**, showing the off-set bilayers.

TBC6 crystallized from ethyl acetate forms crystals without any solvent molecules (**11**). In contrast, the TBC6 molecules form chains through interactions between dimers II (−119.0 kJ mol^−1^, Table 1 ▸) and III (−73.2 kJ mol^−1^), ultimately yielding a 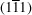 bilayer [Fig. 10 ▸(*a*)]. Although both electrostatic and dispersion interactions play roles, the latter make the major contribution to the total interaction energy. The off-set bilayer fits on top of another bilayer, shifted by half a molecule, forming dimer VII (−120.9 kJ mol^−1^), where a ^*t*^Bu group of the *A*-type ring also fits into the cavity. Between these layers, C—H⋯O interactions occur [C30—H30C⋯O1(−1 + *x*, *y*, *z*) distance of 3.52 (2) Å], forming dimer IX (−51.2 kJ mol^−1^). Packing extends toward the [011] direction via centrosymmetric dimer VIII (−106.1 kJ mol^−1^), where the molecules bind through the ^*t*^Bu group of the *A*-type ring. In addition, the molecules form two C—H⋯π inter­actions with a distance of 3.64 (3) Å [C3⋯C9(1 − *x*, 2 − *y*, 1 − *z*)].

**Figure 11:**
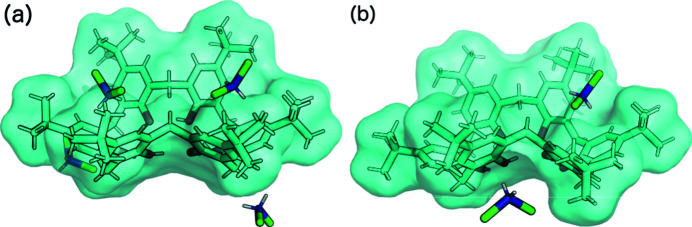
The asymmetric units of TBC6 solvated with DCM: (*a*) 1:4 (**12**) and (*b*) 1:2 (**13**). Disorder has been removed for clarity.

Structures **12** [Fig. 11 ▸(*a*)] and **13** [Fig. 11 ▸(*b*)], with 1:4 and 1:2 ratios, respectively, crystallized from DCM at lower temperatures than structure **3** with the 1:3 ratio. Both structures crystallized in the 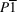 triclinic space group with one molecule of TBC6 in the asymmetric unit.

The crystal structure of TBC6 with four molecules of DCM (**12**) was found to possess some unique packing features. More specifically, the DCM molecules occupy two cavities inside TBC6, both of which are disordered over two positions. The DCM molecules form C—H⋯π contacts [3.626 (4) Å for motif I and 3.653 (5) Å for motif I′], resulting in interaction energies of −59.4 and −52.4 kJ mol^−1^, respectively (Table 1 ▸). In addition, the chlorine atoms point towards the exterior of the binding cavity. This structure also possesses off-set bilayers, which are built through motifs II and III with inter­action energies of −130.7 and −124.6 kJ mol^−1^, respectively. In this case, the second bilayer is only slightly shifted, trapping four molecules of DCM between the host molecules, and the DCM molecules form a channel along the [110] direction that separates the TBC6 bilayer [Fig. 10 ▸(*b*)]. The second channel is formed by TBC6 molecules along the [001] direction, with four DCM molecules being trapped in the capsule. The molecules that are outside the capsules are located at different positions to those in previous structures. More specifically, both DCM molecules are located near the *B*-type phenyl rings, with interaction energies of −15.0 and −14.6 kJ mol^−1^. Some similarities were observed with the aceto­nitrile-containing structure (2:3, VARGOL); however, only one aceto­nitrile molecule is occupied inside TBC6, with an interaction energy of −57.5 6 kJ mol^−1^, and a second one is positioned outside the cavity, with an interaction energy of −55.2 kJ mol^−1^. In addition, an off-set bilayer is present, built by motifs II and III; however, here interactions between two bilayers are formed through motifs VI and VII with interaction energies of −54.4 and −129.6 kJ mol^−1^, respectively, whereas in structure **12** they are formed through DCM molecules.

Structure **13** is formed slowly from an undersaturated DCM solution at room temperature. In this structure, the TBC6 molecules are again packed in an off-set bilayer that is built through motifs II and VI (Fig. 4 ▸). Dimer VI is formed by the shifting of one molecule to form a π–π stacking, with a distance between atoms C11 and C17(2 − *x*, −*y*, −*z*) of 3.194 (3) Å and an interaction energy of −68.3 kJ mol^−1^. One of the binding cavities is occupied by the DCM molecules, which form C—H⋯π interactions (motif I). The second cavity is filled with ^*t*^Bu groups from molecules in the neighbouring layer (motif VII), resulting in a total interaction energy of −123.1 kJ mol^−1^. The same ratio was observed in the structure containing aceto­nitrile (VARGUR 1:2), in which both guest molecules occupy TBC6 cavities. Consequently, the ^*t*^Bu group lies above the aceto­nitrile molecule and the interaction energy of motif VII is higher (−40.8 kJ mol^−1^).

### 3.6. Fill percentage of the host cavity

**Figure 12:**
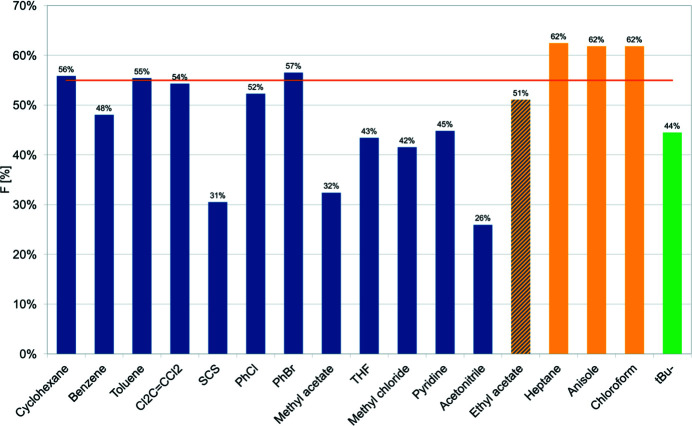
Fill percentages (*F*) for the various guest molecules (for the major component if disorder is present in the crystal structure). Blue denotes guest molecules that form inclusion complexes, yellow denotes guests that form exclusion complexes and cross-hatching indicates ethyl acetate from which the unsolved crystal structure is obtained. Green represents the volume of the ^*t*^Bu group. The orange line corresponds to *F* = 55%.

In the pinched-cone conformation of TBC6, there are two small cavities surrounded by two *B*-type rings and one *A*-type ring. Each cavity has an average volume of 175 Å^3^, as determined for each crystal structure using the *Voidoo* software (Kleywegt & Jones, 1994 ▸). The calculated fill percentages *F* of the guest molecules are presented in Fig. 12 ▸. This analysis was also extended to crystal structures found for TBC6 in the CSD that form host–guest interactions, namely complexes with tetra­chloro­ethene (Andreetti *et al.*, 1989 ▸), carbon di­sulfide (Schatz, Backes &Siehl, 2000 ▸), chloro­benzene (Ramon *et al.*, 2011 ▸), bromo­benzene (Ramon *et al.*, 2011 ▸) and aceto­nitrile (Dale *et al.*, 2003 ▸). For the guests that form inclusion complexes, an *F* value of 55% appears to be the upper limit, with many complexes having lower values. The majority of guest molecules are disordered over two positions, with the exceptions of cyclo­hexane, toluene, tetra­chloro­ethene and carbon di­sulfide, the first three of which have nearly optimal *F* values. Although chloro­benzene and bromo­benzene have *F* values close to 55%, disorder is still present in their crystal structures. Moreover, there is no direct correlation between the guest size and the cavity geometry. The ^*t*^Bu group has an *F* parameter of 45% (Fig. 12 ▸), thereby rendering it a good partner for self-inclusion with TBC6, in addition to promoting the formation of strong dimers with energy lower than −80 kJ mol^−1^ (motifs IV, VII, and VIII). This interaction energy is lower than the interaction energy of motif I (Table 1 ▸)

The distance between the methyl­ene groups that point towards the TBC6 cavity (the *S* distance, denoted by green lines in Fig. 2 ▸) for most structures was close to 4.8 (2) Å (Table S25). More extreme values were observed for DCM molecules in structures possessing different ratios, the shortest being in structure **3** [4.265 (8) Å] and the longest being in structures **9** [anisole, 5.239 (7) Å] and **13** [DCM, 1:2, 5.109 (3) Å]. These longer distances are probably due to the presence of structural motif VII in the crystal packing, since the ^*t*^Bu group from the *B*-type ring occupies one of the cavities of the macrocycle.

**Figure 13:**
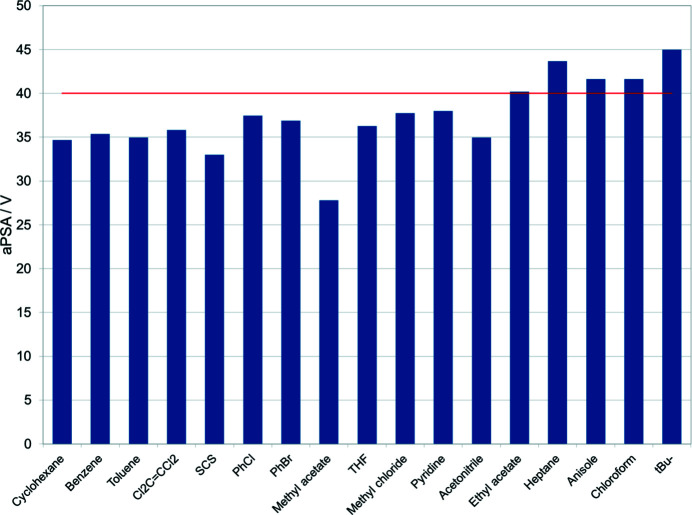
aPSA/*V* values for the various guest molecules. The final column indicates the corresponding value for the ^*t*^Bu group. The red line corresponds to aPSA/*V* = 40.

Although ethyl acetate can, in terms of volume, fit into the binding pocket of TBC6, crystallization from this solvent produced only pure TBC6 crystals. Furthermore, larger guests, such as heptane and anisole (volumes > 100 Å^3^), were found to form exclusion complexes (Fig. 12 ▸). Thus, the consideration of volume alone cannot explain why ethyl acetate does not form an inclusion complex. Therefore, the various properties of the guest molecules (*e.g.* surface area, apolar surface area, polar surface area and dipole moment) and their combinations were evaluated. Although the surface area can explain the ability to form inclusion or exclusion complexes, it does not explain the behaviour of ethyl acetate. However, a parameter that considers both properties together, *i.e.* the ratio of the apolar surface area to the volume (aPSA/*V*), can explain all observed binding modes (Fig. 13 ▸). The value of this parameter for ethyl acetate is considered a boundary, with guest mol­ecules possessing aPSA/*V* ratios lower than 40 forming inclusion complexes, and those with ratios higher than 40 forming exclusion complexes.

## 4. Conclusions

This study constitutes the first extensive investigation of the energetic features of the solvated crystal structures of simple *p-tert*-butyl­calix[6]arene (TBC6) complexes. In general, this work confirms the previously reported affinity of TBC6 for solvents and their inclusion in its crystal network. The different packing schemes found for the solvated crystal structures suggest that specific intermolecular interactions between TBC6 and selected solvents are crucial in the aggregation and crystallization processes. Consequently, these interactions determine the structures and compositions of the final products.

A common structural feature of most host–guest structures is off-set bilayer packing, which is built by the strongest dimers between TBC6 which have energies lower than −110 kJ mol^−1^. This structural feature was also present in pure TBC6 crystallized from ethyl acetate. The incorporation of solvent molecules with volumes <100 Å^3^ leads to separation of the off-set bilayers, whereas larger solvent molecules prevent the formation of this layer in the crystal structures.

The preferred structure crystallizes in the monoclinic *P*2_1_/*c* space group with one guest molecule occupying the TBC6 cavity. Isostructural crystals with a host–guest ratio of 1:1 were observed for TBC6 crystallized from cyclo­hexane, benzene, toluene, methyl acetate, pyridine, tetra­hydro­furan, carbon di­sulfide, bromo­benzene, chloro­benzene and tetra­chloro­ethyl­ene. In contrast, a higher host–guest ratio of 1:3 was observed for crystallization at higher temperatures, which gave isostructural crystal solvates with benzene and pyridine, and a similar structure with di­chloro­methane (DCM). In addition, different stoichiometries were obtained for small guest molecules such as DCM (1:2 and 1:4) and aceto­nitrile (2:3, 1:2).

All guest molecules that occupy the TBC6 cavity interact with the host with an energy close to −50 kJ mol^−1^; therefore, this property does not determine molecular recognition. Even though the formation of a structural motif with the *tert*-butyl group in the TBC6 cavity is energetically favoured, its slow rate of formation possibly limits the formation of pure TBC6 crystals.

Evaluation of the *F* parameter (the fill percentage) shows that apolar molecules occupy approximately 55% of the cavity, whereas more polar molecules have cavity occupancies as low as 32%, without the formation of any strong contacts. Generally, a lower *F* value leads to disorder in the binding or/and a wider range of possible crystal packing that is controlled by the crystallization temperature.

The ratio of the apolar surface area to the volume (aPSA/*V*) was found to be suitable for predicting the formation of exclusion or inclusion complexes, with a boundary of 40 corresponding to the non-solvated crystal structure obtained with ethyl acetate. Therefore, this study allows us to predict whether or not a molecule can bind to the calixarene cavity, and envisages a possible crystal packing scheme that is crucial in the contexts of supramolecular chemistry, gas sorption and host–guest complex formation. Guest-molecule exchange between solution and crystal structure is currently being researched.

## Supplementary Material

Crystal structure: contains datablock(s) global, x6_heptane_10, 11_x6_ethyl_acetate, 12_x6_DCM_1_4, 13_x6_DCM_1_2, 1_x6_benzene, x6_DCM_13_3, 4_x6_cyclohexane, 5_x6_toluene, x6_methyl_acetate, 7_x6_THF, 9_x6_anisole. DOI: 10.1107/S2052252521010678/ed5024sup1.cif

Structure factors: contains datablock(s) 1_x6_benzene. DOI: 10.1107/S2052252521010678/ed5024-1-benzenesup2.hkl

Structure factors: contains datablock(s) x6_DCM_13_3. DOI: 10.1107/S2052252521010678/ed5024-3-DCM-1-3sup3.hkl

Structure factors: contains datablock(s) 4_x6_cyclohexane. DOI: 10.1107/S2052252521010678/ed5024-4-cyclohexanesup4.hkl

Structure factors: contains datablock(s) 5_x6_toluene. DOI: 10.1107/S2052252521010678/ed5024-5-toluenesup5.hkl

Structure factors: contains datablock(s) x6_methyl_acetate. DOI: 10.1107/S2052252521010678/ed5024-6-methylacetatesup6.hkl

Structure factors: contains datablock(s) 7_x6_THF. DOI: 10.1107/S2052252521010678/ed5024-7-THFsup7.hkl

Structure factors: contains datablock(s) 9_x6_anisole. DOI: 10.1107/S2052252521010678/ed5024-9-anisolesup8.hkl

Structure factors: contains datablock(s) x6_heptane_10. DOI: 10.1107/S2052252521010678/ed5024-10-heptanesup9.hkl

Structure factors: contains datablock(s) 11_x6_ethyl_acetate. DOI: 10.1107/S2052252521010678/ed5024-11-ethylacetatesup10.hkl

Structure factors: contains datablock(s) x6_DCM_1_4. DOI: 10.1107/S2052252521010678/ed5024-12-DCM-1-4sup11.hkl

Structure factors: contains datablock(s) x6_DCM_1_2. DOI: 10.1107/S2052252521010678/ed5024-13-DCM-1-2sup12.hkl

Additional tables and figures. DOI: 10.1107/S2052252521010678/ed5024sup13.pdf

CCDC references: 2073896, 2073898, 2073900, 2073902, 2073913, 2073914, 2073919, 2073924, 2073927, 2073929, 2073946

## Figures and Tables

**Table 1:** Total interaction energies (kJ mol^−1^) for selected host–guest (I–I′′′) and host–host (II–X) structural motifs in the analysed crystals calculated in *CrystalExplorer* using the Hartree–Fock method with the 3-21G basis set

			Motif											
Structure	Guest	Ratio	I	I′	I′′	I′′′	II	III	IV	V	VI	VII	VIII	IX
**1**	Benzene	1:3	−52.3	−49.6	−23.8		−138.0	−113.6						
**2**	Pyridine	1:3	−50.8	−6.9	−5.0		−128.9	−115.3						
**3**	DCM	1:3	−62.1	−52.3	−17.7		−124.4†							
**4**	Cyclo­hexane	1:1	−50.4				−132.1	−103.8	−81.6	−20.3				
**5**	Toluene	1:1	−48.7				−138.6	−101.1	−81.1	−19.6				
**6**	Methyl acetate	1:1	−39.1				−138.4	−107.3	−86.5	−21.4				
**7**	THF	1:1	−49.4				−136.1	−110.4	−89.9	−23.7				
**8**	Pyridine	1:1	−57.1				−134.2	−110.4	−83.2	−21.4				
**KENBUA**	Cl_2_C=CCl_2_	1:1	−29.0				−126.6	−112.2	−78.3	−13.0				
**LODNIB**	CS_2_	1:1	−24.5				−127.7	−105.7	−75.3	−14.2				
**UWIVUS**	PhCl	1:1	−57.9				−132.0	−98.5	−79.3	−19.4				
**UWIWAZ**	PhBr	1:1	−19.9				−141.2	−61.7	−80.7	−15.5				
**9**	Anisole	1:1				−33.6				−27.1	−90.5	−118.6‡		
**10**	*n*-Heptane	1:1				−39.5			−84.7§	−10.5	−92.6			
**11**	–						−119.0	−73.2				−120.9	−106.1	−51.2
**12**	DCM	1:4	−59.4	−52.4	−15.0¶	−14.6¶	−130.7	−124.6						
**VERGOL**	MeCN	2:3	−57.5			−55.2	−115.2	−77.2			−54.4	−129.6		
**13**	DCM	1:2	−52.3			−37.1	−112.4				−68.3	−123.1		
**VARGUR**	MeCN	1:2	−54.1	−42.5			−110.2††					−40.8		−35.9

† Structure **3** has two motifs II.

‡ Structure **9** has two motifs VII, the second of which has a total interaction energy of −111.7 kJ mol^−1^.

§ Structure **10** has two motifs IV, the second of which has a total interaction energy of −68.9 kJ mol^−1^.

¶ The DCM molecules are situated close to the *B* ring.

†† The VARGUR structure has two motifs II.

**Table 2:** Interaction metrics stabilizing inclusion compounds (motif I)

Structure	Type	*D*—H⋯*A*	*D*⋯*A* (Å)	*D*—H⋯*A* (°)
**4**	C—H⋯π	C67—H67⋯C38	3.634 (6)	158.5
**5**	C—H⋯π	C71—H71⋯C35	3.679 (8)	141.5
**6**	C=O⋯π	O7*A*⋯C17	3.17 (2)	
**7**	C—H⋯π	C67*A*—H67*C*⋯C48	3.86 (2)	−161
**8**	C—H⋯π	C69—H69⋯C58	3.760 (5)	−161.2
**KENBUA**	Cl⋯π	Cl3⋯C61	3.40	
**LODNIB**	S⋯π	S1⋯C8	3.592 (6)	
**UWIVUS**	C—H⋯π	C69—H69⋯C30	3.630 (7)	140.7
**UWIWAZ**	C—H⋯π	C74—H74⋯C17	3.58 (2)	168.0
