# Supplementary material for: Insights into molecular recognition from the crystal structures of p-tert-butyl­calix[6]arene complexed with different solvents
Source: IUCrJ. 2021 Nov 16;9(Pt 1):55–64. doi: 10.1107/S2052252521010678 (PMC8733874; doi:10.1107/S2052252521010678)
Supplement: Supplementary file 13 [file m-09-00055-sup13.pdf]

IUCrJ

Volume 9 (2022)

Supporting information for article:

Insights into molecular recognition from the crystal structures of *p-tert*-butylcalix[6]arene complexed with different solvents

Maura Malinska

Insights into Molecular Recognition from the Crystal Structures of p-tert-Butylcalix[6]arene Complexed with Different Solvents

Maura Malinska*

Faculty of Chemistry, University of Warsaw, Poland

E-mail: mmalinska@chem.uw.edu.pl

Phone: +48 22 55 26356

List of Figures

S1 Energy frameworks generated for the **1** crystal 12

S2 Energy frameworks generated for the **2** crystal 13

S3 Energy frameworks generated for the **3** crystal 14

S4 Energy frameworks generated for the **4** crystal 15

S5 Energy frameworks generated for the **5** crystal 16

S6 Energy frameworks generated for the **6** crystal 17

S7 Energy frameworks generated for the **7** crystal 18

S8 Energy frameworks generated for the **8** crystal 19

S9 Energy frameworks generated for the **9** crystal 20

S10 Energy frameworks generated for the **10** crystal 21

S11 Energy frameworks generated for the **11** crystal 22

S12 Energy frameworks generated for the **12** crystal 23

S13 Energy frameworks generated for the **13** crystal 24

List of Tables

S1 Crystallization condition summary 3

S2 XRD data colections for **1**, **3–5** 5

S3 XRD data collections for **6**, **7**, **9**, **10** 6

S4 XRD data collections for **11– 13** 7

S5 Interaction Energies for Selected Dimer Motifs (I-III) Observed in **1** Crystal Structure[a] 12

S6 Interaction Energies for Selected Dimer Motifs (I-III) Observed in **2** Crystal Structure[a] 15

S7 Interaction Energies for Selected Dimer Motifs (I-III) Observed in **3** Crystal Structure[a] 16

S8 Interaction Energies for Selected Dimer Motifs (I-V) Observed in **4** Crystal Structure[a] 17

S9 Interaction Energies for Selected Dimer Motifs (I-V) Observed in **5** Crystal Structure[a] 18

S10 Interaction Energies for Selected Dimer Motifs (I-V) Observed in **6** Crystal Structure[a] 19

S11 Interaction Energies for Selected Dimer Motifs (I-V) Observed in **7** Crystal Structure[a] 20

S12 Interaction Energies for Selected Dimer Motifs (I-V) Observed in **8** Crystal Structure[a] 21

S13 Interaction Energies for Selected Dimer Motifs (I-V) Observed in **UWIVUS** Crystal Structure[a] 22

S14 Interaction Energies for Selected Dimer Motifs (I-V) Observed in **UWIWAZ** Crystal Structure[a] 23
S15 Interaction Energies for Selected Dimer Motifs (I-V) Observed in **KENBUA** Crystal Structure[a] 24
S16 Interaction Energies for Selected Dimer Motifs (I-V) Observed in **LODNIB** Crystal Structure[a] 25
S17 Interaction Energies for Selected Dimer Motifs Observed in **9** Crystal Structure[a] 25
S18 Interaction Energies for Selected Dimer Motifs Observed in **10** Crystal Structure[a] 25
S19 Interaction Energies for Selected Dimer Motifs Observed in **11** Crystal Structure[a] 26
S20 Interaction Energies for Selected Dimer Motifs Observed in **12** Crystal Structure[a] 26
S21 Interaction Energies for Selected Dimer Motifs Observed in **13** Crystal Structure[a] 26
S22 Interaction Energies for Selected Dimer Motifs Observed in **VARGUR** Crystal Structure[a] 27
S23 Interaction Energies for Selected Dimer Motifs Observed in **VARGOL** Crystal Structure[a] 27
S24 Interaction energy between host-guest calculated by B3LYP-D3/6-31G** method in Gaussian16 28
S25 The **S** distance for TBC6 molecule in the **1** – **13** crystal structures 28

Table S1: Crystallization condition summary

Structure	solvent	TBC6 [mg]	solvent [mL]	T [deg C]
1	benzene	4.9	0.6	55
2	DCM	4.0	0.5	66
4	cyclohexane	4.0	0.5	25
5	toluene	4.2	0.4	25
6	methyl acetate	4.7	1.0	25
7	THF	10.6	1.2	66
9	anisole	9.4	0.5	50
10	n-heptane	10.0	0.8	55
11	ethyl acetate	4.2	1.0	70
12	DCM	5.1	4.0	30
13	DCM	3.2	4.0	30

Crystallization

X-ray Diffraction Experiments

Structure solution and refinement details

Crystal structure 1 (benzene 1:3)

Crystal was a non-merohedral twin with c.a. 1:1 ratio. Component 2 is rotated by 180deg around [0.71 0.71 0.0] axis in the reciprocal space. One of the three benzene molecules is disordered over two position with occupancy factors of 0.53(1) and 0.47(1). As some bond distance in benzene ring with atom labels C79-C84 were too long, therefore we applied the AFIX66 constraint. In addition, one of the six tert-butyl groups of the calixarene reveals a twofold positional disorder with site occupancy factors 0.86(1) and 0.14(1). To ensure a proper shape of ADPs the ISOR, DELU and SIMU restraints and proper geometry the DFIX and DANG restraints were applied for disorder tert-butyl group. The H atoms were positioned geometrically and allowed to ride on their parent atoms, with O–H = 0.84Å, C–H = 0.95–0.99Å and Uiso(H) = 1.2–1.5 Ueq (parent atom). The highest density peak is located close to atom C83 in the disordered benzene ring.

Table S2: XRD data colections for **1**, **3–5**

	1_x6_benzene	3_x6_DCM_1_3	4_x6_cyclohexane	5_x6_toluene
Crystal data				
Chemical formula	$C_{66}H_{84}O_{6}3\,(C_6H_6)$	$C_{66}H_{84}O_{6}3\,(CH_2Cl_2)$	$C_{66}H_{84}O_6C_6H_{12}$	$C_{66}H_{84}O_6C_7H_8$
Mr	1207.65	1228.1	1057.48	1065.46
Crystal system, space group	Triclinic, P-1	Monoclinic, P21/c	Monoclinic, P21/n	Monoclinic, P21/n
a, b, c (Å)	13.5391(8), 14.7702(6), 19.045(1)	17.8792(10), 19.6559(15), 18.9669(10)	17.2109(4), 18.2986(4), 19.6172(4)	17.1817(6), 18.7393(6), 19.4157(6)
α, β, γ (deg)	80.680(4), 72.393(5), 78.191(4)	90, 96.732(5), 90	90, 94.521(2), 90	90, 95.843(3), 90
V (Å^3)	3532.8(3)	6619.6(7)	6158.9(2)	6218.9(3)
Z	2	4	4	4
Radiation type	CuKα	MoKα	CuKα	CuKα
μ (mm^{-3})	0.54	0.31	0.54	0.54
Crystal size (mm)	0.32 x 0.12 x 0.08	0.36 x 0.12 x 0.05	0.16 x 0.10 x 0.08	0.1 x 0.09 x 0.04
Data collection				
Diffractometer	SuperNova, Dual, Cu at home/near, Atlas	SuperNova, Dual, Cu at zero, Atlas	SuperNova, Dual, Cu at zero, Atlas	SuperNova, Dual, Cu at zero, Atlas
Absorption correction	Multi-scan	Multi-scan	Multi-scan	Multi-scan
Tmin, Tmax	0.906, 1.000	0.52, 1.00	0.669, 1.000	0.896, 1.000
No. of measured, independent and observed [I > 2σ(I)] reflections	23648, 23648, 17758	42673, 13524, 7357	19018, 19018, 11945	11419, 5095, 4242
Rint	twin	0.119	twin	0.029
Θ_{max} (deg)	70.1	26.4	71.7	46.9
(sin Θ/λ)max (Å^{-1})	0.61	0.625	0.616	0.473
Refinement				
R[F2 > 2σ(F2)], wR(F2), S	0.046, 0.129, 1.03	0.119, 0.335, 1.06	0.065, 0.197, 1.05	0.065, 0.167, 1.23
No. of reflections	23648	13520	19018	5095
No. of parameters	892	781	803	799
No. of restraints	60	35	77	6
$\Delta\rho_{max}$, $\Delta\rho_{min}$ (e/Å^{-3})	0.34, -0.36	0.74, -0.72	0.63, -0.42	0.38, -0.28

Table S3: XRD data collections for **6**, **7**, **9**, **10**

	6_x6_methyl_acetate	7_x6_THF	9_x6_anisole	10_x6_nheptane
Crystal data				
Chemical formula	$C_{66}H_{84}O_6C_3H_6O_2$	$C_{66}H_{84}O_6C_4H_8O$	$C_{66}H_{84}O_6C_7H_8O$	$C_{66}H_{84}O_6C_7H_{16}$
Mr	1047.4	1045.43	1081.46	1073.5
Crystal system, space group	Monoclinic, P21/n	Monoclinic, P21/n	Triclinic, P-1	Monoclinic, P21/c
a, b, c (Å)	17.0366(3), 18.4996(3), 19.7620(3)	17.1261(4), 17.8832(4), 19.8489(5)	14.1349(8), 16.1763(11), 17.0355(10)	16.2520(5), 23.5373(7), 17.2102(4)
α, β, γ (*deg*)	90, 94.288(2), 90	90, 93.914(2), 90	83.979(5), 72.392(5), 73.292(5)	90, 101.773(3), 90
V (Å^3)	6210.96(18)	6064.9(2)	3555.3(4)	6444.9(3)
Z	4	4	2	[illegible]
Radiation type	CuKα	CuKα	MoKα	CuKα
μ (mm^{-1})	0.56	0.56	0.06	0.5
Crystal size (mm)	0.20 x 0.13 x 0.09	0.24 x 0.15 x 0.11	0.2 x 0.17 x 0.1	0.24 x 0.13 x 0.05
Data collection				
Diffractometer	SuperNova, Dual, Cu at zero, Atlas	SuperNova, Dual, Cu at zero, Atlas	Xcalibur, Opal	SuperNova, Dua Cu at home/near Atlas
Absorption correction	Multi-scan	Multi-scan	Multi-scan	Multi-scan
Tmin, Tmax	0.881, 1.000	0.705, 1.000	0.841, 1.000	0.870, 1.000
No. of measured, independent and observed [I > 2σ(I)] reflections	35347, 11386, 8785	21493, 21493, 14854	49801, 13019, 6833	37071, 12820, 6910
Rint	0.032	twin	0.102	0.06
Θ_{max} (deg)	69.1	77.1	25.4	74.
(sin $\Theta/\lambda)_{max}$ (Å^{-1})	0.606	0.632	0.602	0.62
Refinement				
R[F2 > 2σ(F2)], wR(F2), S	0.083, 0.238,1.07	0.079, 0.242, 1.08	0.143, 0.437, 1.36	0.066, 0.207, 1.04
No. of reflections	11386	21493	13019	1282
No. of parameters	825	874	762	80
No. of restraints	16	169	132	8
$\Delta\rho_{max}$, $\Delta\rho_{min}$ (e/Å^{-3})	0.28, -0.48	0.47, -0.35	2.13, -0.87	0.71, -0.43

Table S4: XRD data collections for **11**– **13**

	11_x6_ethyl_acetate	12_x6_DCM_1_4	13_x6_DCM_1_2
Crystal data			
Chemical formula	$C_{66}H_{84}O_6$	$C_{66}H_{84}O_{64}(CH_2Cl_2)$	$C_{66}H_{84}O_6 2\,(CH_2Cl_2)$
Mr	973.33	1313.03	1143.18
Crystal system, space group	Triclinic, P-1	Triclinic, P-1	Triclinic, P-1
a, b, c (Å)	9.5900(6), 18.199(7), 18.547(8)	13.8727(6), 16.0717(6), 17.6177(6)	12.2610(7), 17.2425(11), 17.4170(12)
α, β, γ (deg)	114.31(4), 91.36(2), 91.70(2)	77.127(3), 66.915(4), 76.404(3)	118.625(7), 92.647(5), 98.062(5)
V (Å^3)	2946.5(19)	3474.5(3)	3172.8(4)
Z	2	2	2
Radiation type	Cu Ka	Cu Ka	Cu Ka
μ (mm^{-1})	0.53	3.34	2.08
Crystal size (mm)	0.67 x 0.07 x 0.02	0.15 x 0.13 x 0.1	0.3 x 0.2 x 0.1
Data collection			
Diffractometer	SuperNova, Dual, Cu at zero, Atlas	SuperNova, Dual, Cu at zero, Atlas	SuperNova, Dual, Cu at zero, Atlas
Absorption correction	Multi-scan	Multi-scan	Multi-scan
Tmin, Tmax	0.831, 1.000	0.599, 1.000	0.506, 1.000
No. of measured, independent and observed [I $>$ 2σ(I)] reflections	7563, 6064, 2856	40412, 13310, 10893	37155, 12208, 9900
Rint	0.07	0.057	0.039
Θ_{max} (deg)	58.9	71.7	71.7
(sin Θ/λ)max (Å^{-1})	0.556	0.616	0.616
Refinement			
R[F2 $>$ 2σ(F2)], wR(F2), S	0.156, 0.440, 1.19	0.072, 0.222, 1.02	0.051, 0.142, 1.02
No. of reflections	7289	13310	12208
No. of parameters	694	808	766
No. of restraints	97	0	3
$\Delta\rho_{max}$, $\Delta\rho_{min}$	0.54, -0.40	0.94, -1.20	0.31, -0.72

Crystal structure 3 (DCM 1:3)

One of the three DCM molecules is disordered over two position with occupancy factors of 0.72(1) and 0.28(1). This DCM molecule occupy void, that allows for a movement. Only two positions were refined, however restraints for C-Cl bond and Cl...Cl distance were applied to ensure proper geometry of the molecule. In addition, three of the six tert-butyl groups of the calixarene needed the DFIX and DANG restraints to ensure proper geometry. The H atoms were positioned geometrically and allowed to ride on their parent atoms, with O–H = 0.84Å, C–H = 0.95–0.99Å and Uiso(H) = 1.2–1.5 Ueq (parent atom). The highest density peak is located close to atom Cl6 in the disordered DCM molecule.

Crystal structure 4 (cyclohexane 1:1)

Crystal was a non-merohedral twin with major component of 0.70(1). Component 2 is rotated by 180deg around [0.0 0.0 1.0]. The H atoms were positioned geometrically and allowed to ride on their parent atoms, with O–H = 0.84Å, C–H = 0.95–0.99Å and Uiso(H) = 1.2–1.5 Ueq (parent atom). Three of the six tert-butylgroups of the calixarene reveals a twofold positional disorder with site occupancy factors for first position of 0.65(1), 0,51(1), and 0.78(1), respectively. The highest density peak is located at atom C31. Disordered tert-butyl groups were refined with geometrical restraints (DFIX and DANG). The cyclohexane bond length was restrained to 1.54(1)Å.

Crystal structure 5 (toluene 1:1)

Two of the six tert-butyl groups of the calixarene reveals a twofold positional disorder with site occupancy factors for first position of 0.61(1) and 0.79(1). The highest density peak is located at atom C31. Disordered tert-butyl groups were refined with geometrical restraints (DFIX and DANG). As some ADPs parameters were unreasonable, the SIMU and ISOR restraints were applied for disordered tert butyl groups. The H atoms were positioned geometrically and allowed to ride on their parent atoms, with O–H = 0.84Å, C–H = 0.95–0.99Å

and Uiso(H) = 1.2–1.5 Ueq (parent atom).

Crystal structure 6 (methyl acetate 1:1)

Methyl acetate molecule is disordered over two positions with occupancy factors 0.86(<1) and 0.14(<1). Similar geometry parameters were enforced by SADI restraints. In addition FLAT restraint for non-H atom for both conformations of methyl acetate was used. Three of the six tert-butyl groups of the calixarene reveals a twofold positional disorder with site occupancy factors for first position of 0.61(<1), 0.51(<1) and 0.88(<1). The highest density peak is located at atom C31. Disordered tert-butyl groups were refined with geometrical restraints (DFIX and DANG). As some ADPs parameters were unreasonable, the SIMU and ISOR restraints were applied for disordered tert-butyl groups. The H atoms were positioned geometrically and allowed to ride on their parent atoms, with O–H = 0.84Å, C–H = 0.95–0.99Å and Uiso(H) = 1.2–1.5 Ueq (parent atom).

Crystal structure 7 (THF 1:1)

Crystal was a non-merohedral twin with major component of 0.521(1). Component 2 is rotated by 180deg around [0.0 0.0 1.0]. THF molecule is disordered over two positions with occupancy factors 0.64(<1) and 0.14(<1). Geometry parameters were restrained with DFIX and DANG. As some ADPs were unstable during refinement, SIMU and DELU retraints were used. Four of the six tert-butyl groups of the calixarene reveals a twofold positional disorder with site occupancy factors for first position of 0.61(<1), 0.86(<1), 0.76(<1), and 0.53(<1). The highest density peak is located at atom C8A. Disordered tert-butyl groups were refined with geometrical restraints (DFIX and DANG). As some ADPs parameters were unreasonable, the SIMU and DELU restraints were applied for disordered tert-butyl groups. The atom C63, C64, C65 were constrained to be identical with atom C63A, C64A and C65A, as non positively defined ADPs were observed for this tert butyl group. The H atoms were positioned geometrically and allowed to ride on their parent atoms, with O–H = 0.84Å, C–H

= 0.95–0.99Å and Uiso(H) = 1.2–1.5 Ueq (parent atom).

Crystal structure 9 (anisole 1:1)

Anisole molecule is disordered over two positions with occupancy factors 0.91(<1) and 0.09(<1). Low quality od XRD data (low I/σ and high Rint) unable to stable refinement of anisole molecule, therefore rigid body of anisole with AFIX 5 was used. As some ADPs were unstable during refinement, we applied two sets of EADP constraints for aromatic ring and methoxy group. The highest density peak is located at atom C67 of anisole molecule. Four of the six tert-butyl groups of the calixarene reveals a twofold positional disorder with site occupancy factors for first position of 0.66(<1), 0.84(<1), 0.74(<1), and 0.68(<1). Disordered tert-butyl groups were refined with geometrical restraints (DFIX and DANG). As some ADPs parameters were unreasonable, the ISOR restraints were applied for disordered tert-butyl groups. Some atoms from disordered tert-butyl groups were constraint to have the same ADPs, as non positively definite ADPs were observed. The H atoms were positioned geometrically and allowed to ride on their parent atoms, with O–H = 0.84Å, C–H = 0.95–0.99Å and Uiso(H) = 1.2–1.5 Ueq (parent atom).

Crystal structure 10 (heptane 1:1)

Heptane molecule were refined with C-C distance restraint to 1.54Å. The highest density peak is located at atom C68 of heptane molecule. Three of the six tert-butyl groups of the calixarene reveals a twofold positional disorder with site occupancy factors for first position of 0.49(<1), 0.53(<1), and 0.60(<1). Disordered tert-butyl groups were refined with geometrical restraints (DFIX and DANG). As some ADPs parameters were unreasonable, the ISOR, SIMU and DELU restraints were applied for disordered tert-butyl groups. Some atoms from disordered tert-butyl groups were constraint to have the same ADPs, as non positively definite ADPs were observed. The H atoms were positioned geometrically and allowed to ride on their parent atoms, with O–H = 0.84Å, C–H = 0.95–0.99Å and Uiso(H)

= 1.2–1.5 Ueq (parent atom).

Crystal structure 11 (ethyl acetate)

Weak diffraction from monocrystal resulted in low statistics (Rint=7.13% and I/σ=6.7) and low completeness of 68%. One of the six tert-butyl groups of the calixarene reveals a twofold positional disorder with site occupancy factors for first position of 0.52(<1). Disordered tert-butyl group was refined with geometrical restraints (DFIX and DANG). As some ADPs parameters were unreasonable, the ISOR, RIGU restraints were applied for disordered tert-butyl group. Some atoms from disordered tert-butyl groups were constraint to have the same ADPs n(C8 and C8A), as non positively definite ADPs were observed. The H atoms were positioned geometrically and allowed to ride on their parent atoms, with O–H = 0.84Å, C–H = 0.95–0.99Å and Uiso(H) = 1.2–1.5 Ueq (parent atom).

Crystal structure 12 (DCM 1:4)

Two of the four DCM molecules are disordered over two position with occupancy factors of 0.63(1) and 0.68(1). The DCM molecules were refined with restraints for C-Cl bond and Cl...Cl distance to ensure proper geometry of the molecule. In addition, two of the six tert-butyl groups of the calixarene reveals a two fold positional disorder with site occupancy factors for first position of 0.92(<1) and 0.812(<1). The tert-butyl groups were refined with DFIX and DANG restraints to ensure proper geometry. The H atoms were positioned geometrically and allowed to ride on their parent atoms, with O–H = 0.84Å, C–H = 0.95–0.99Å and Uiso(H) = 1.2–1.5 Ueq (parent atom).

Crystal structure 13 (DCM 1:2)

Two DCM molecules are disordered over two position with occupancy factors of 0.74(<1) and 0.89(<1). The DCM molecules were refined with restraints for C-Cl bond and Cl...Cl distance to ensure proper geometry of the molecule. Also ISOR restraint were applied to

obtain reasonable ADPs. In addition, one of the six tert-butyl groups of the calixarene reveals a two fold positional disorder with site occupancy factors for first position of 0.81(<1). The tert-butyl groups were refined with DFIX and DANG restraints to ensure proper geometry. The H atoms were positioned geometrically and allowed to ride on their parent atoms, with O–H = 0.84Å, C–H = 0.95–0.99Å and Uiso(H) = 1.2–1.5 Ueq (parent atom). The highest density peak is located close to atom Cl1 in the disordered DCM molecule.

Crystal Framework Analysis

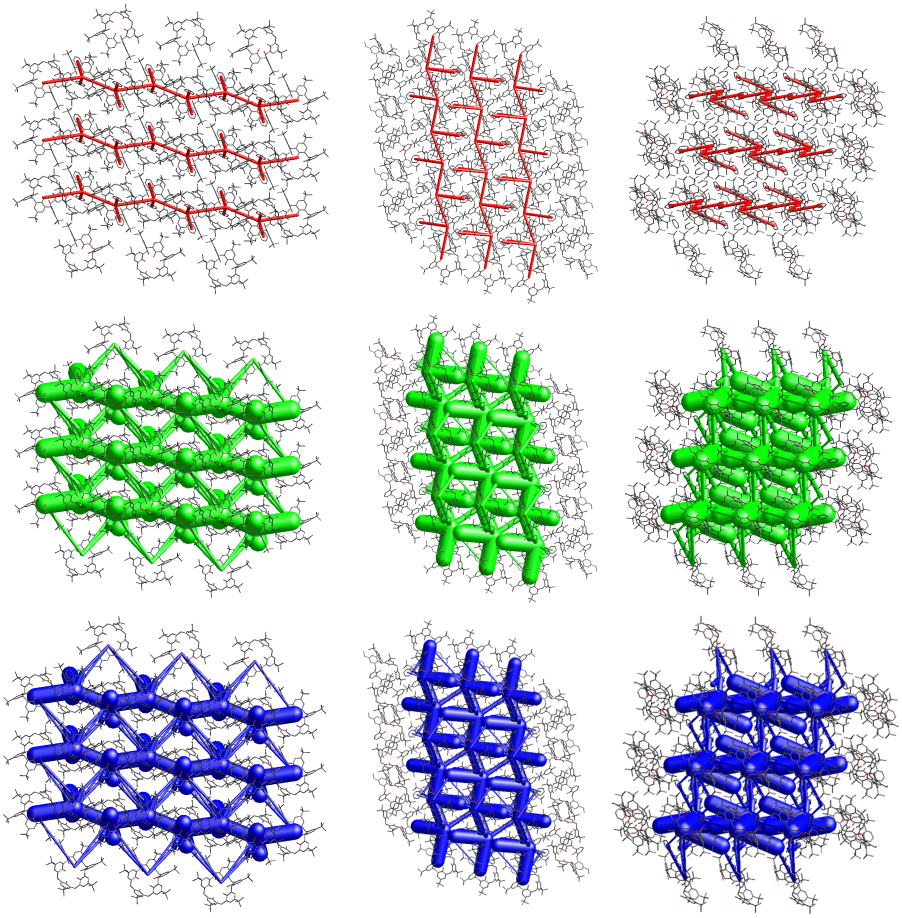

Figure S1: Energy frameworks generated for the **1** crystal. Line thickness indicates the interaction energy value (the thicker the line the greater the energy). In rows electrostatic energy (red), dispersion energy (green), and total energy (blue). Views are presented along the [100] axis (1st column), [010] axis (2nd column) and [001] axis (3rd column))

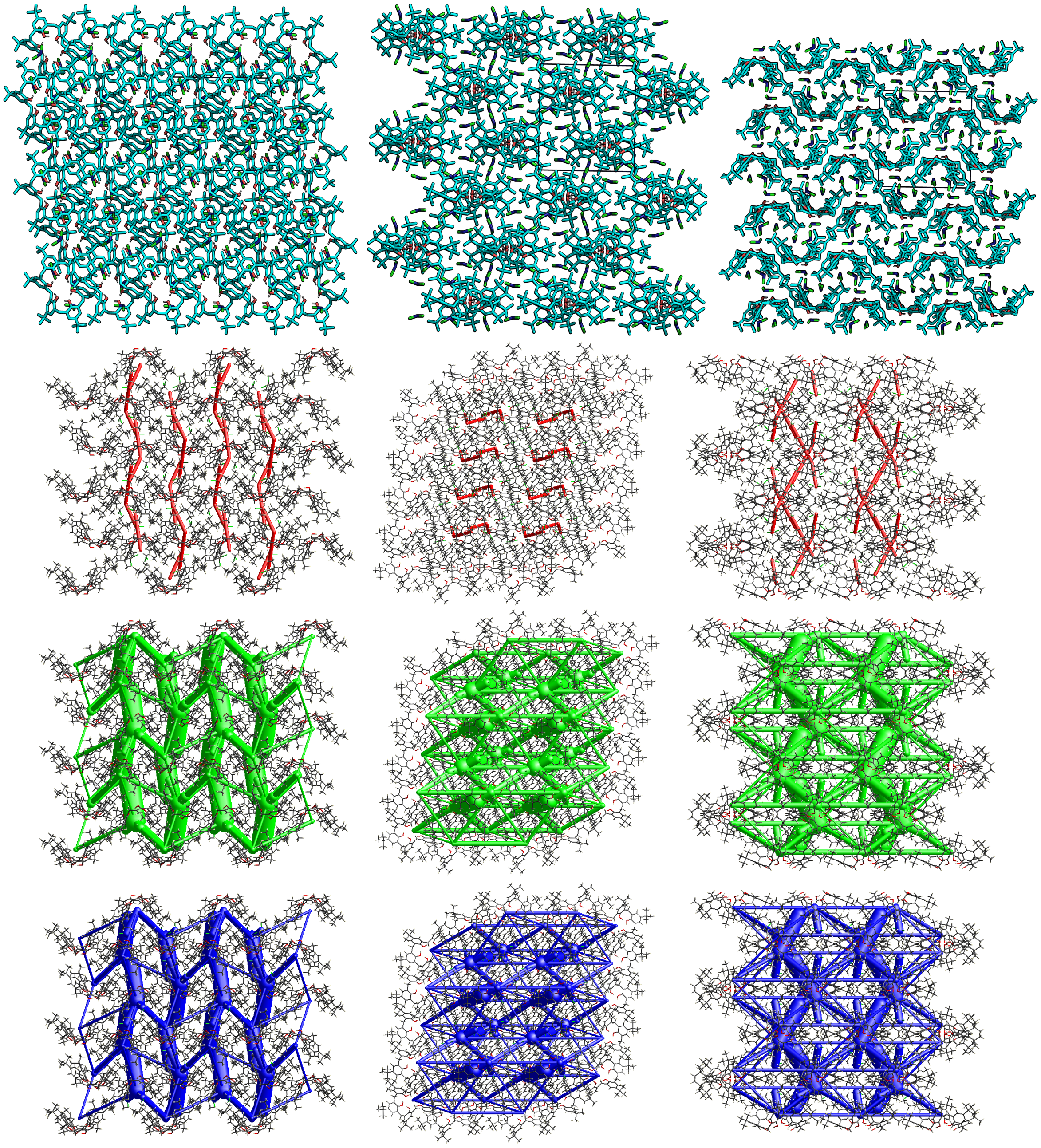

Figure S2: Energy frameworks generated for the **2** crystal. Line thickness indicates the interaction energy value (the thicker the line the greater the energy). In rows electrostatic energy (red), dispersion energy (green), and total energy (blue). Views are presented along the [100] axis (1st column), [010] axis (2nd column) and [001] axis (3rd column))

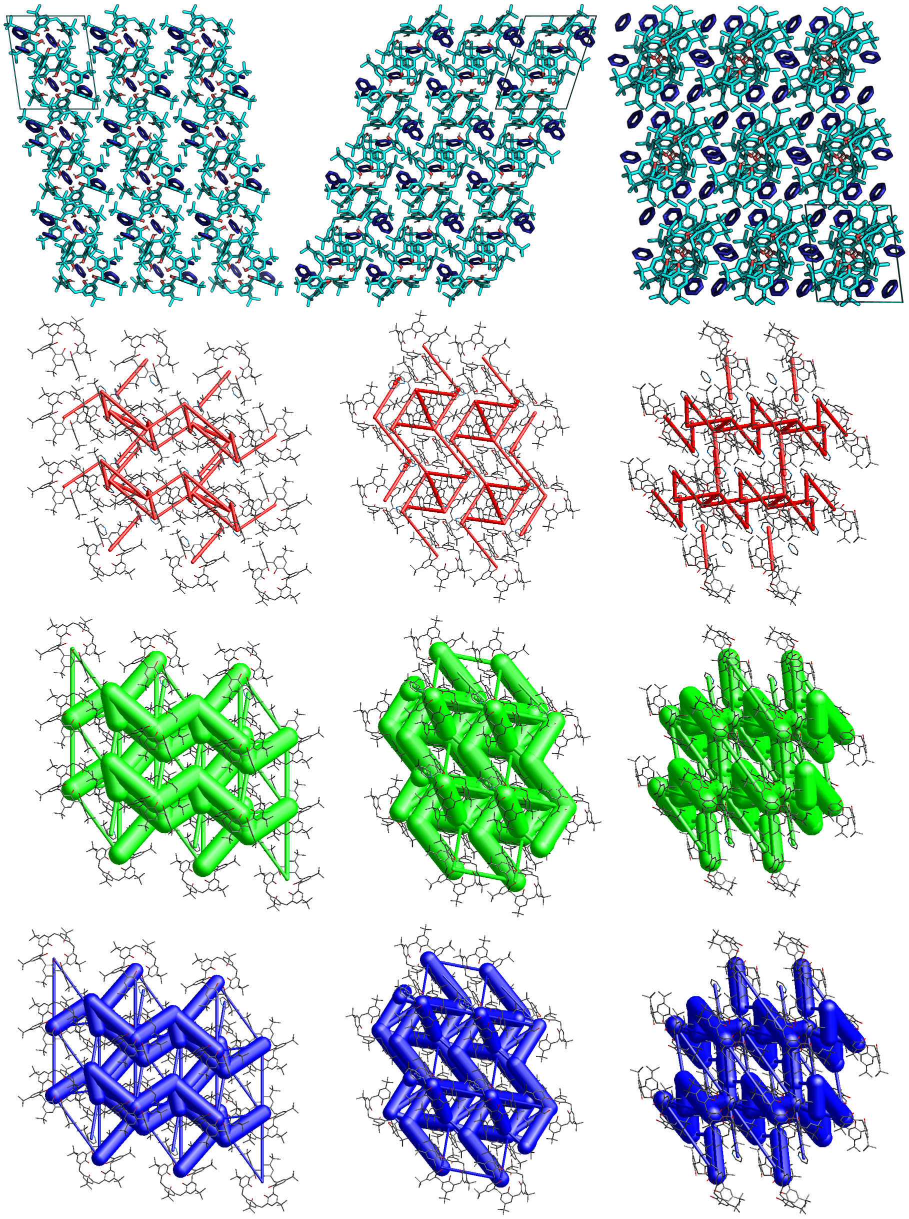

Figure S3: Energy frameworks generated for the **3** crystal. Line thickness indicates the interaction energy value (the thicker the line the greater the energy). In rows electrostatic energy (red), dispersion energy (green), and total energy (blue). Views are presented along the [100] axis (1st column), [010] axis (2nd column) and [001] axis (3rd column))

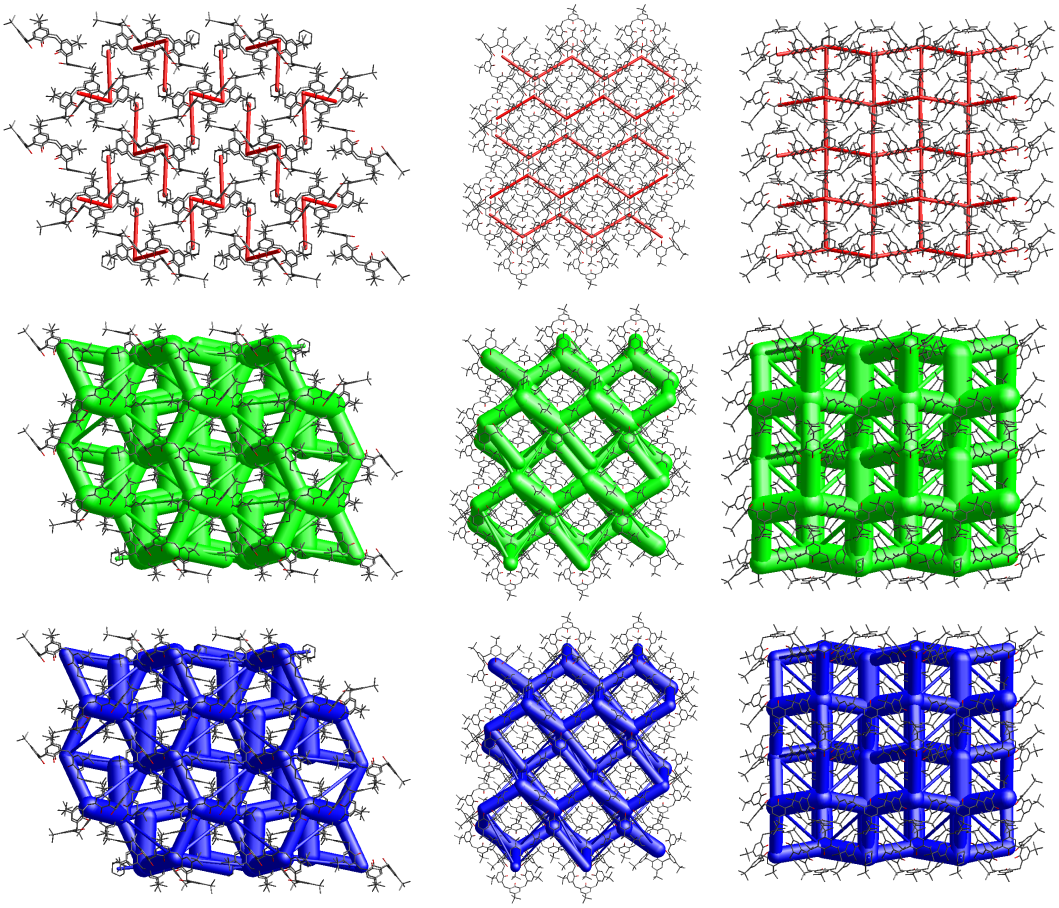

Figure S4: Energy frameworks generated for the **4** crystal. Line thickness indicates the interaction energy value (the thicker the line the greater the energy). In rows electrostatic energy (red), dispersion energy (green), and total energy (blue). Views are presented along the [100] axis (1st column), [010] axis (2nd column) and [001] axis (3rd column))

Table S5: Interaction Energies for Selected Dimer Motifs (I-III) Observed in **1** Crystal Structure[a]

motif	E_{ele}^{CE}	$E_{pol}^{CE}1$	E_{dis}^{CE}	E_{rep}^{CE}	E_{tot}^{CE}
I	-14.2	-4.9	-73.3	38.7	-52.3
I'	-17.6	-5.1	-73.3	46.9	-49.6
I"	-7.8	-1.9	-26.4	11.3	-23.8
II	-26.1	-10.4	-189.3	81.3	-138.0
III	-25.1	-8.4	-167.1	83.9	-113.6

[a] E^{CE} represents the interaction energies computed with CrystaExplorer. E_{ele}^{CE}, E_{pol}^{CE}, E_{dis}^{CE}, E_{rep}^{CE} and E_{tot}^{CE} are the electrostatic, polarization, dispersive, repulsive, and total interaction energies, respectively.

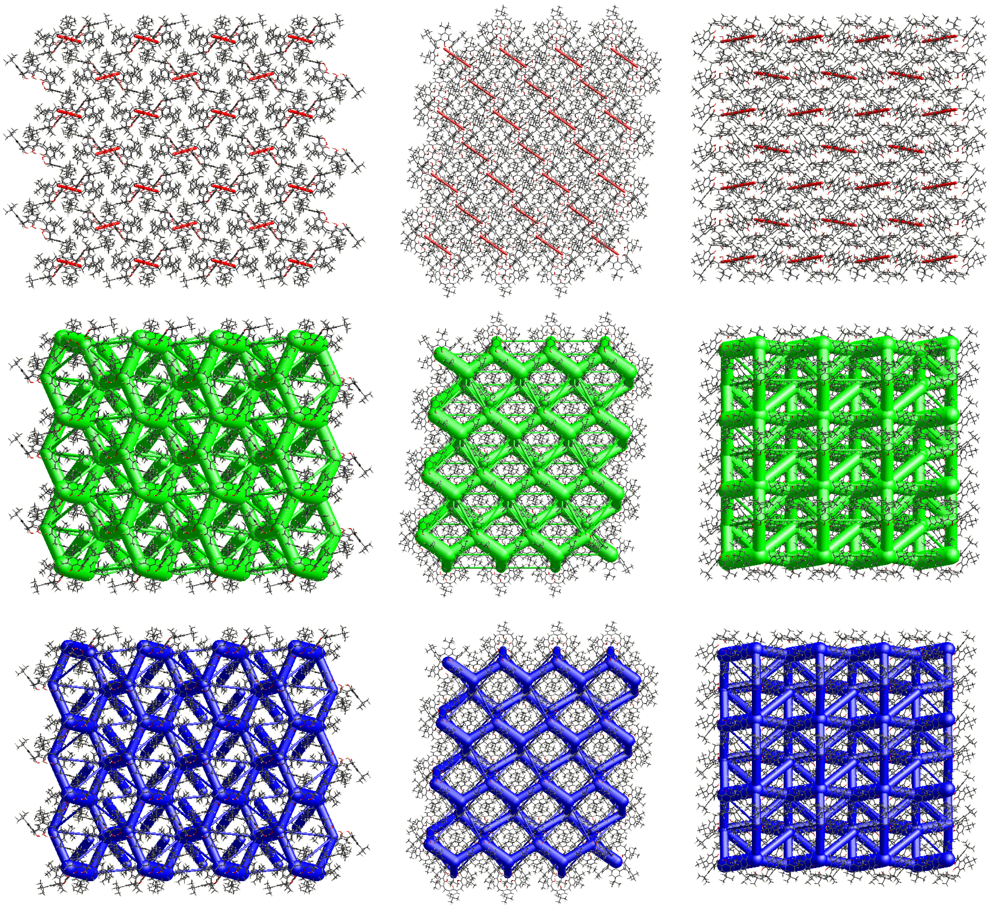

Figure S5: Energy frameworks generated for the **5** crystal. Line thickness indicates the interaction energy value (the thicker the line the greater the energy). In rows electrostatic energy (red), dispersion energy (green), and total energy (blue). Views are presented along the [100] axis (1st column), [010] axis (2nd column) and [001] axis (3rd column))

Table S6: Interaction Energies for Selected Dimer Motifs (I-III) Observed in **2** Crystal Structure[a]

motif	E^{CE}_{ele}	E^{CE}_{pol}	E^{CE}_{dis}	E^{CE}_{rep}	E^{CE}_{tot}
I	-5.2	-1.3	-74.2	27.4	-50.8
I'	-0.5	-0.3	-7.0	0.1	-6.9
I"	0.7	-0.3	-6.2	0.0	-5.0
II	-20.0	-9.9	-174.6	68.1	-128.9
III	-26.2	-8.8	-165.8	82.1	-115.3

[a] E^{CE} represents the interaction energies computed with CrystaExplorer. E^{CE}_{ele}, E^{CE}_{pol}, E^{CE}_{dis}, E^{CE}_{rep} and E^{CE}_{tot} are the electrostatic, polarization, dispersive, repulsive, and total interaction energies, respectively.

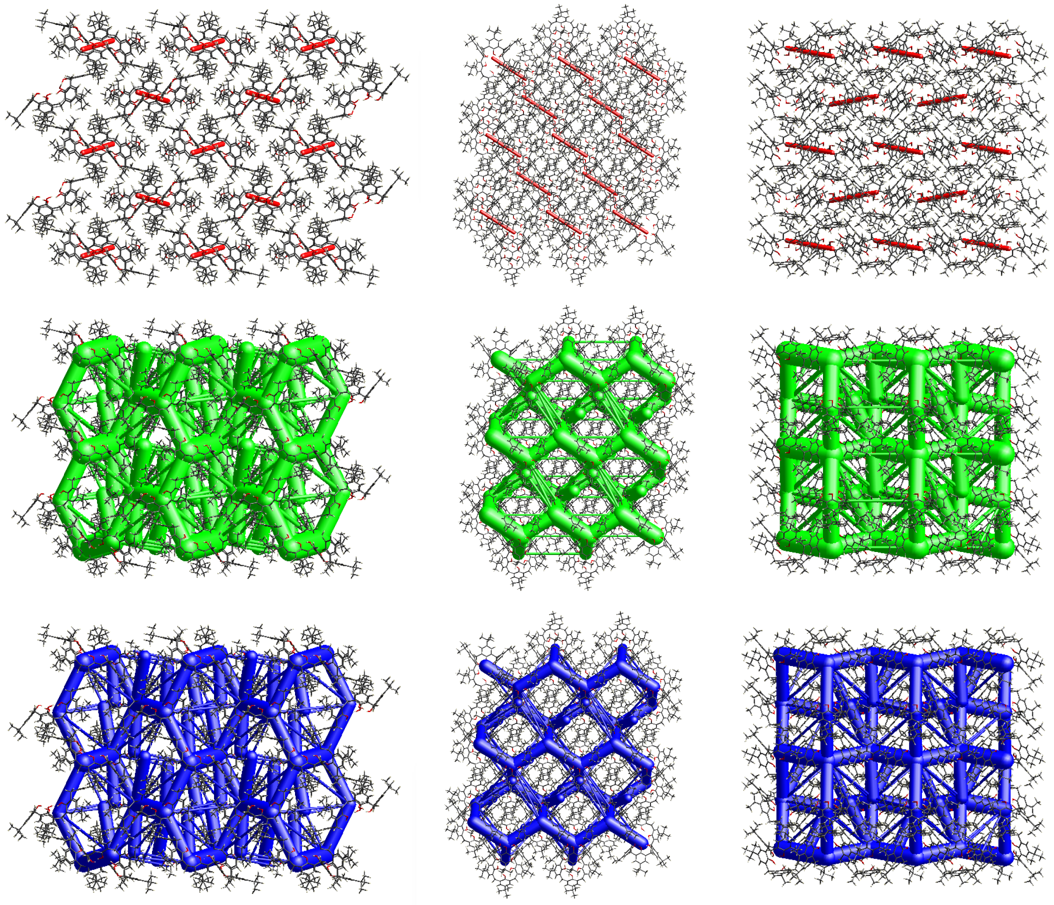

Figure S6: Energy frameworks generated for the **6** crystal. Line thickness indicates the interaction energy value (the thicker the line the greater the energy). In rows electrostatic energy (red), dispersion energy (green), and total energy (blue). Views are presented along the [100] axis (1st column), [010] axis (2nd column) and [001] axis (3rd column))

Table S7: Interaction Energies for Selected Dimer Motifs (I-III) Observed in **3** Crystal Structure[a]

motif	E^{CE}_{ele}	E^{CE}_{pol}	E^{CE}_{dis}	E^{CE}_{rep}	E^{CE}_{tot}
I	-25.2	-7.5	-54.9	22.1	-62.1
I'	-19.2	-7.7	-58.8	31.0	-52.3
I"	-3.1	-2.1	-27.0	13.7	-17.7
II	-18.1	-11.8	-158.6	55.2	-124.4

[a] E^{CE} represents the interaction energies computed with CrystaExplorer. E^{CE}_{ele}, E^{CE}_{pol}, E^{CE}_{dis}, E^{CE}_{rep} and E^{CE}_{tot} are the electrostatic, polarization, dispersive, repulsive, and total interaction energies, respectively.

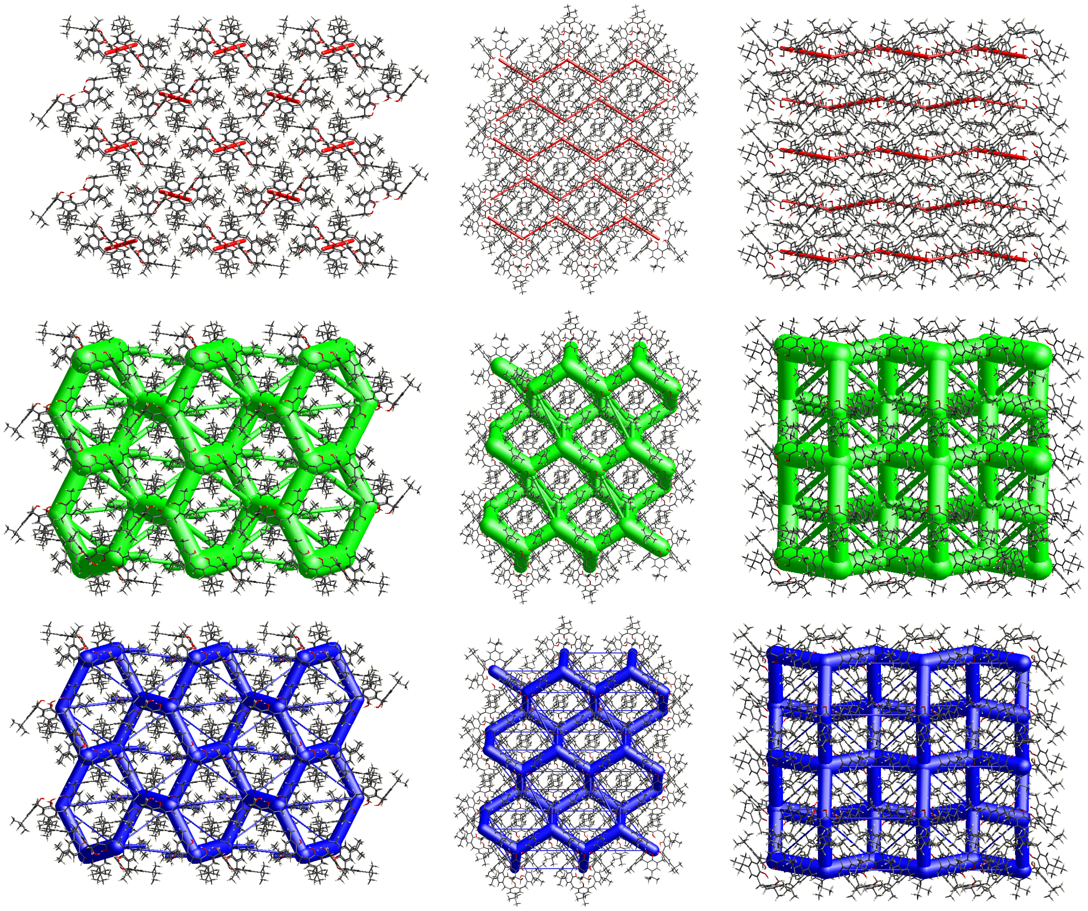

Figure S7: Energy frameworks generated for the **7** crystal. Line thickness indicates the interaction energy value (the thicker the line the greater the energy). In rows electrostatic energy (red), dispersion energy (green), and total energy (blue). Views are presented along the [100] axis (1st column), [010] axis (2nd column) and [001] axis (3rd column))

Table S8: Interaction Energies for Selected Dimer Motifs (I-V) Observed in **4** Crystal Structure[a]

motif	E_{ele}^{CE}	E_{pol}^{CE}	E_{dis}^{CE}	E_{rep}^{CE}	E_{tot}^{CE}
I	-9.9	-3.3	-74.7	35.9	-50.4
II	-22.0	-9.5	-186.7	79.2	-132.1
III	-23.7	-10.9	-151.5	78.8	-103.8
IV	-14.4	-5.2	-131.7	67.9	-81.6
V	-2.7	-0.3	-32.6	14.8	-20.3

[a] E^{CE} represents the interaction energies computed with CrystaExplorer. E_{ele}^{CE}, E_{pol}^{CE}, E_{dis}^{CE}, E_{rep}^{CE} and E_{tot}^{CE} are the electrostatic, polarization, dispersive, repulsive, and total interaction energies, respectively.

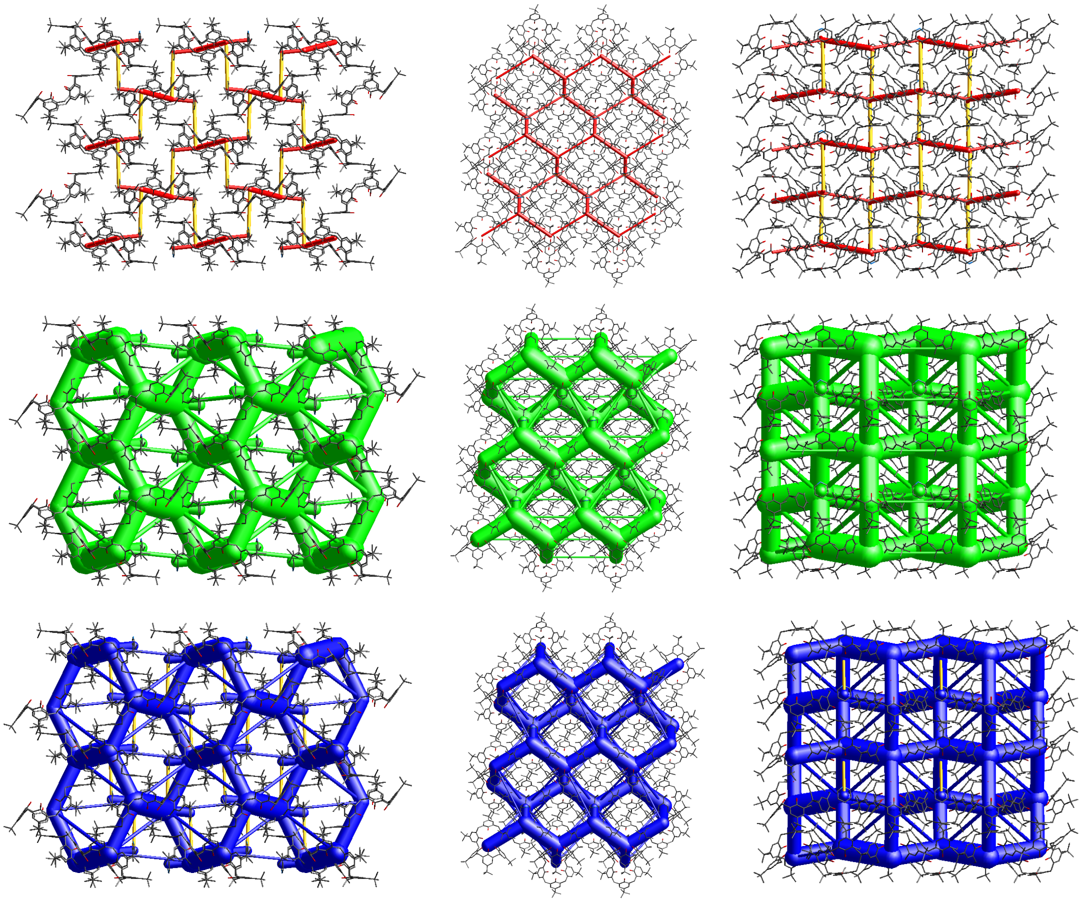

Figure S8: Energy frameworks generated for the 2 crystal. Line thickness indicates the interaction energy value (the thicker the line the greater the energy). In rows electrostatic energy (red), dispersion energy (green), and total energy (blue). Views are presented along the [100] axis (1st column), [010] axis (2nd column) and [001] axis (3rd column))

Table S9: Interaction Energies for Selected Dimer Motifs (I-V) Observed in **5** Crystal Structure[a]

motif	E^{CE}_{ele}	E^{CE}_{pol}	E^{CE}_{dis}	E^{CE}_{rep}	E^{CE}_{tot}
I	-6.7	-4.6	-71.6	31.6	-48.7
II	-25.5	-10.7	-187.4	78.1	-138.6
III	-14.6	-8.0	-137.8	53.2	-101.1
IV	-13.1	-5.8	-126.2	61.3	-81.1
V	-2.3	-0.2	-30.9	13.1	-19.6

[a] E^{CE} represents the interaction energies computed with CrystaExplorer. E^{CE}_{ele}, E^{CE}_{pol}, E^{CE}_{dis}, E^{CE}_{rep} and E^{CE}_{tot} are the electrostatic, polarization, dispersive, repulsive, and total interaction energies, respectively.

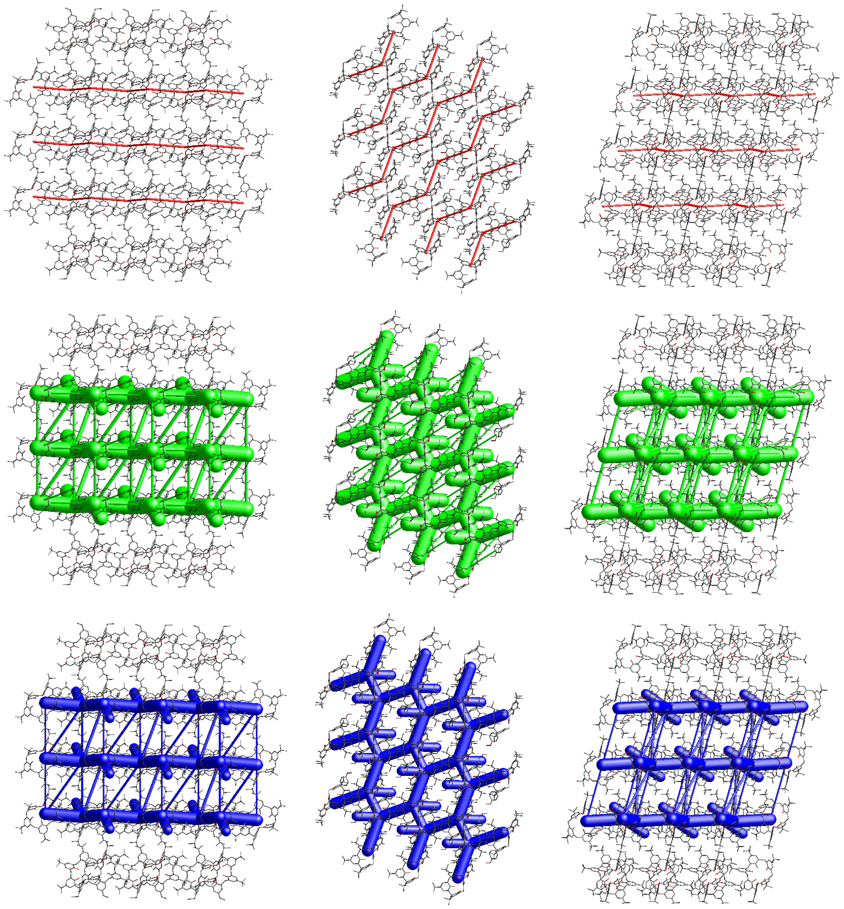

Figure S9: Energy frameworks generated for the **9** crystal. Line thickness indicates the interaction energy value (the thicker the line the greater the energy). In rows electrostatic energy (red), dispersion energy (green), and total energy (blue). Views are presented along the [100] axis (1st column), [010] axis (2nd column) and [001] axis (3rd column))

Table S10: Interaction Energies for Selected Dimer Motifs (I-V) Observed in **6** Crystal Structure[a]

motif	E_{ele}^{CE}	E_{pol}^{CE}	E_{dis}^{CE}	E_{rep}^{CE}	E_{tot}^{CE}
I	-4.5	-2.6	-47.1	11.7	-39.3
II	-18.7	-9.2	-168.7	47.4	-138.4
III	-13.4	-7.7	-133.6	39.2	-107.3
IV	-8.2	-5.1	-115.6	36.2	-86.5
V	-5.4	-0.2	-38.4	23.2	-21.4

[a] E^{CE} represents the interaction energies computed with CrystaExplorer. E_{ele}^{CE}, E_{pol}^{CE}, E_{dis}^{CE}, E_{rep}^{CE} and E_{tot}^{CE} are the electrostatic, polarization, dispersive, repulsive, and total interaction energies, respectively.

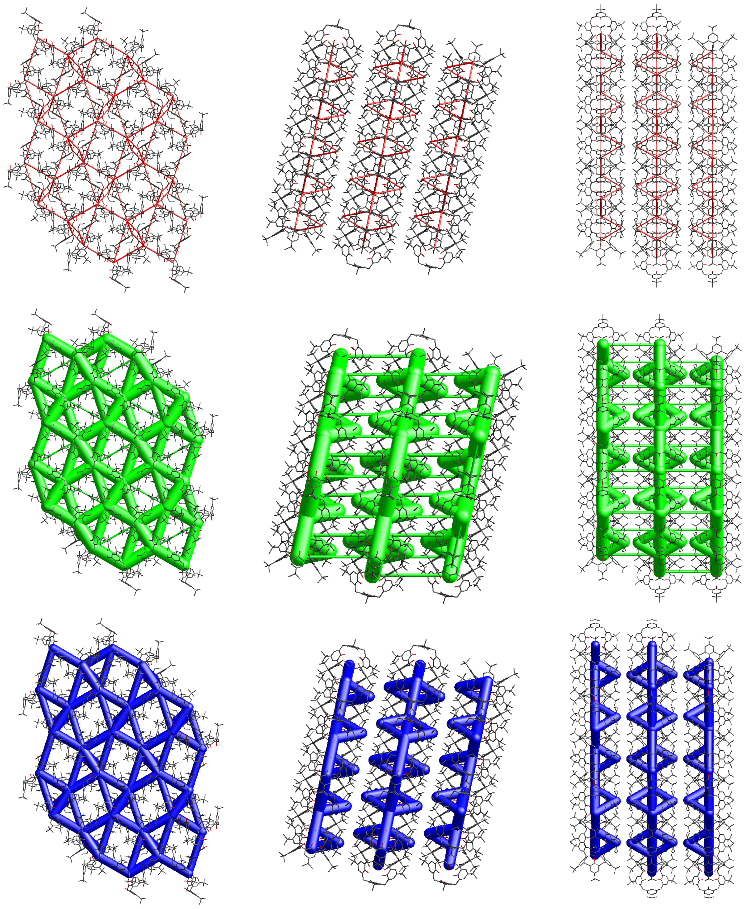

Figure S10: Energy frameworks generated for the **10** crystal. Line thickness indicates the interaction energy value (the thicker the line the greater the energy). In rows electrostatic energy (red), dispersion energy (green), and total energy (blue). Views are presented along the [100] axis (1st column), [010] axis (2nd column) and [001] axis (3rd column))

Table S11: Interaction Energies for Selected Dimer Motifs (I-V) Observed in **7** Crystal Structure[a]

motif	E^{CE}_{ele}	E^{CE}_{pol}	E^{CE}_{dis}	E^{CE}_{rep}	E^{CE}_{tot}
I	-7.4	-3.5	-56.8	14.3	-49.4
II	-20.0	-9.9	-184.4	70.1	-136.1
III	-16.4	-8.4	-143.7	50.8	-110.4
IV	-7.3	-5.5	-121.5	37.7	-89.9
V	-1.7	-0.2	-33.4	10.2	-23.7

[a] E^{CE} represents the interaction energies computed with CrystaExplorer. E^{CE}_{ele}, E^{CE}_{pol}, E^{CE}_{dis}, E^{CE}_{rep} and E^{CE}_{tot} are the electrostatic, polarization, dispersive, repulsive, and total interaction energies, respectively.

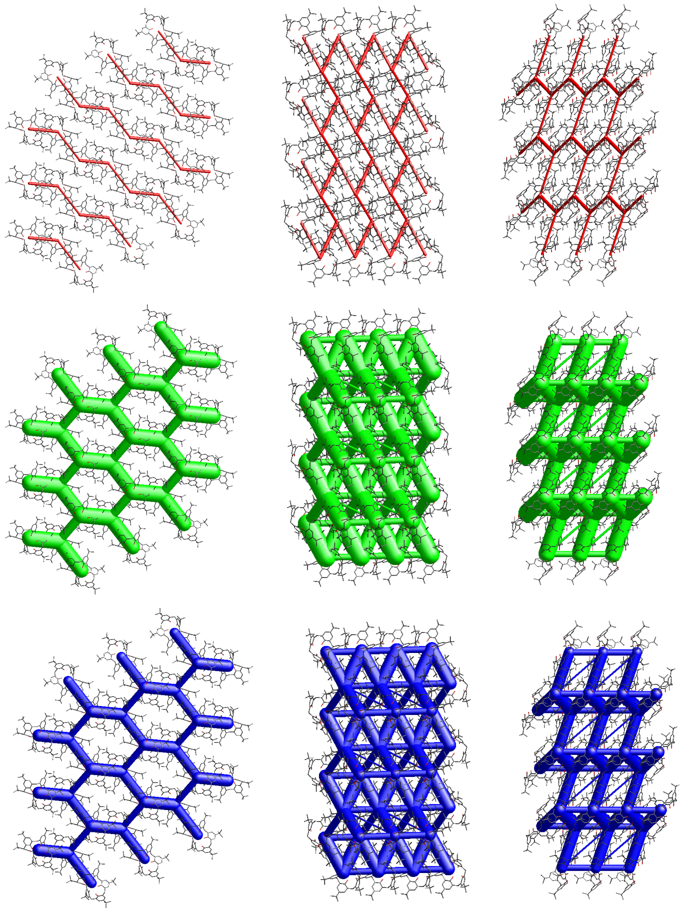

Figure S11: Energy frameworks generated for the **11** crystal. Line thickness indicates the interaction energy value (the thicker the line the greater the energy). In rows electrostatic energy (red), dispersion energy (green), and total energy (blue). Views are presented along the [100] axis (1st column), [010] axis (2nd column) and [001] axis (3rd column))

Table S12: Interaction Energies for Selected Dimer Motifs (I-V) Observed in **8** Crystal Structure[a]

motif	E_{ele}^{CE}	E_{pol}^{CE}	E_{dis}^{CE}	E_{rep}^{CE}	E_{tot}^{CE}
I	-22.9	-6.8	-76.6	48.8	-57.1
II	-32.0	-10.1	-194.4	98.8	-134.2
III	-17.1	-8.2	-149.0	57.6	-110.4
IV	-9.2	-5.5	-127.2	54.8	-83.2
V	-2.1	-0.4	-32.2	12.4	-21.4

[a] E^{CE} represents the interaction energies computed with CrystaExplorer. E_{ele}^{CE}, E_{pol}^{CE}, E_{dis}^{CE}, E_{rep}^{CE} and E_{tot}^{CE} are the electrostatic, polarization, dispersive, repulsive, and total interaction energies, respectively.

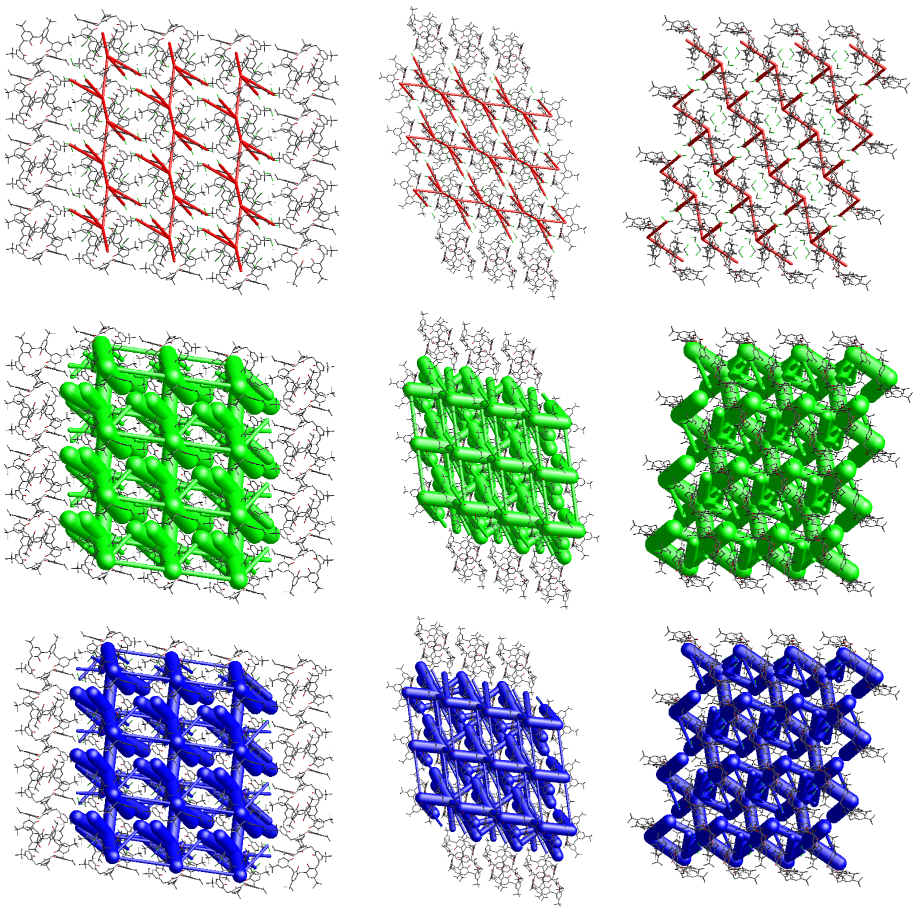

Figure S12: Energy frameworks generated for the **12** crystal. Line thickness indicates the interaction energy value (the thicker the line the greater the energy). In rows electrostatic energy (red), dispersion energy (green), and total energy (blue). Views are presented along the [100] axis (1st column), [010] axis (2nd column) and [001] axis (3rd column))

Table S13: Interaction Energies for Selected Dimer Motifs (I-V) Observed in **UWIVUS** Crystal Structure[a]

motif	E_{ele}^{CE}	E_{pol}^{CE}	E_{dis}^{CE}	E_{rep}^{CE}	E_{tot}^{CE}
I	-19.1	-6.5	-76.6	43.1	-57.9
II	-20.3	-8.9	-178.5	68.3	-132.0
III	-19.8	-7.0	-141.1	65.8	-98.5
IV	-10.2	-5.0	-117.7	49.8	-79.3
V	-1.4	-0.2	-27.8	8.8	-19.4

[a] E^{CE} represents the interaction energies computed with CrystaExplorer. E_{ele}^{CE}, E_{pol}^{CE}, E_{dis}^{CE}, E_{rep}^{CE} and E_{tot}^{CE} are the electrostatic, polarization, dispersive, repulsive, and total interaction energies, respectively.

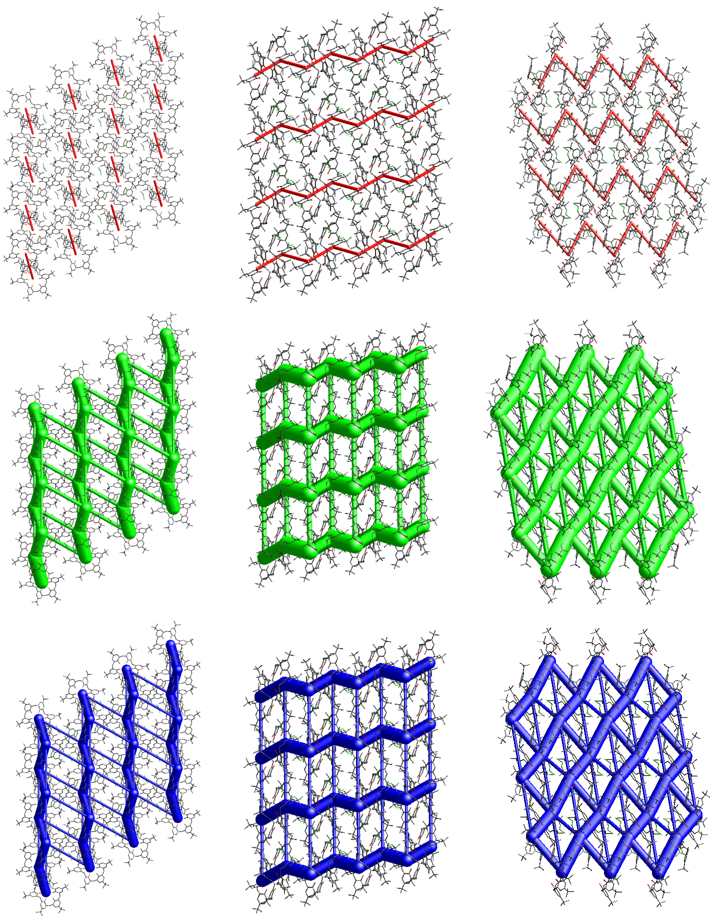

Figure S13: Energy frameworks generated for the **13** crystal. Line thickness indicates the interaction energy value (the thicker the line the greater the energy). In rows electrostatic energy (red), dispersion energy (green), and total energy (blue). Views are presented along the [100] axis (1st column), [010] axis (2nd column) and [001] axis (3rd column))

Table S14: Interaction Energies for Selected Dimer Motifs (I-V) Observed in **UWIWAZ** Crystal Structure[a]

motif	E^{CE}_{ele}	E^{CE}_{pol}	E^{CE}_{dis}	E^{CE}_{rep}	E^{CE}_{tot}
I	-42.4	-7.8	-86.6	131.2	-19.9
II	-20.5	-26.9	-179.9	73.1	-141.2
III	-5.6	-28.1	-127.2	80.8	-61.7
IV	-10.0	-5.0	-118.3	48.5	-80.7
V	-1.5	-0.1	-25.1	10.7	-15.5

[a] E^{CE} represents the interaction energies computed with CrystaExplorer. E^{CE}_{ele}, E^{CE}_{pol}, E^{CE}_{dis}, E^{CE}_{rep} and E^{CE}_{tot} are the electrostatic, polarization, dispersive, repulsive, and total interaction energies, respectively.

Table S15: Interaction Energies for Selected Dimer Motifs (I-V) Observed in **KENBUA** Crystal Structure[a]

motif	E^{CE}_{ele}	E^{CE}_{pol}	E^{CE}_{dis}	E^{CE}_{rep}	E^{CE}_{tot}
I	-15.2	-3.7	-56.7	49.3	-29.0
II	-17.2	-15.6	-158.5	54.3	-126.6
III	-25.4	-9.4	-120.9	35.4	-112.2
IV	-15.7	-5.7	-119.3	60.3	-78.3
V	-1.6	-0.1	-22.0	7.2	-15.9

[a] E^{CE} represents the interaction energies computed with CrystaExplorer. E^{CE}_{ele}, E^{CE}_{pol}, E^{CE}_{dis}, E^{CE}_{rep} and E^{CE}_{tot} are the electrostatic, polarization, dispersive, repulsive, and total interaction energies, respectively.

Table S16: Interaction Energies for Selected Dimer Motifs (I-V) Observed in **LODNIB** Crystal Structure[a]

motif	E^{CE}_{ele}	E^{CE}_{pol}	E^{CE}_{dis}	E^{CE}_{rep}	E^{CE}_{tot}
I	-5.3	-1.1	-38.2	19.8	-24.5
II	-21.4	-7.6	-162.1	55.3	-127.7
III	-16.1	-13.3	-134.7	50.2	-105.7
IV	-8.2	-4.9	-107.9	41.4	-75.3
V	-1.2	-0.1	-20.0	6.3	-14.2

[a] E^{CE} represents the interaction energies computed with CrystaExplorer. E^{CE}_{ele}, E^{CE}_{pol}, E^{CE}_{dis}, E^{CE}_{rep} and E^{CE}_{tot} are the electrostatic, polarization, dispersive, repulsive, and total interaction energies, respectively.

Table S17: Interaction Energies for Selected Dimer Motifs Observed in **9** Crystal Structure[a]

motif	E^{CE}_{ele}	E^{CE}_{pol}	E^{CE}_{dis}	E^{CE}_{rep}	E^{CE}_{tot}
I''	-6.1	-3.0	-44.2	17.7	-33.6
V	-4.0	-0.8	-42.1	18.8	-27.3
VI	7.8	-12.1	-161.8	68.1	-90.5
VII	-20.2	-7.2	-167.6	71.1	-118.6
VII	-17.0	-6.4	-161.8	68.5	-111.7

[a] E^{CE} represents the interaction energies computed with CrystaExplorer. E^{CE}_{ele}, E^{CE}_{pol}, E^{CE}_{dis}, E^{CE}_{rep} and E^{CE}_{tot} are the electrostatic, polarization, dispersive, repulsive, and total interaction energies, respectively.

Table S18: Interaction Energies for Selected Dimer Motifs Observed in **10** Crystal Structure[a]

motif	E^{CE}_{ele}	E^{CE}_{pol}	E^{CE}_{dis}	E^{CE}_{rep}	E^{CE}_{tot}
I”’	-4.2	-2.0	-58.2	23.0	-39.5
IV	-13.7	-5.8	-130.4	62.2	-84.7
IV	-10.0	-3.7	-109.3	52.1	-68.9
V	-1.2	-0.4	-26.7	13.2	-14.8
VI	3.7	-15.8	-142.0	51.7	-92.6

[a] E^{CE} represents the interaction energies computed with CrystaExplorer. E^{CE}_{ele}, E^{CE}_{pol}, E^{CE}_{dis}, E^{CE}_{rep} and E^{CE}_{tot} are the electrostatic, polarization, dispersive, repulsive, and total interaction energies, respectively.

Table S19: Interaction Energies for Selected Dimer Motifs Observed in **11** Crystal Structure[a]

motif	E^{CE}_{ele}	E^{CE}_{pol}	E^{CE}_{dis}	E^{CE}_{rep}	E^{CE}_{tot}
II	-24.8	-9.5	-170.1	80.9	-119.0
III	-8.8	-5.1	-114.5	52.1	-73.2
VII	-26.4	-8.6	-198.5	111.6	-120.9
VIII	-24.1	-7.8	-184.6	110.8	-106.1
IX	-5.0	-3.5	-63.1	16.0	-51.2

[a] E^{CE} represents the interaction energies computed with CrystaExplorer. E^{CE}_{ele}, E^{CE}_{pol}, E^{CE}_{dis}, E^{CE}_{rep} and E^{CE}_{tot} are the electrostatic, polarization, dispersive, repulsive, and total interaction energies, respectively.

Table S20: Interaction Energies for Selected Dimer Motifs Observed in **12** Crystal Structure[a]

motif	E^{CE}_{ele}	E^{CE}_{pol}	E^{CE}_{dis}	E^{CE}_{rep}	E^{CE}_{tot}
I	-26.9	-9.1	-59.5	34.0	-59.4
I’	-19.1	-6.9	-55.2	26.3	-52.4
I”	-2.6	-1.3	-19.7	7.7	-15.0
I”’	-5.9	-1.7	-11.0	3.1	-14.6
II	-18.0	-10.9	-164.8	53.3	-130.7
III	-16.7	-9.2	-158.0	50.2	-124.6

[a] E^{CE} represents the interaction energies computed with CrystaExplorer. E^{CE}_{ele}, E^{CE}_{pol}, E^{CE}_{dis}, E^{CE}_{rep} and E^{CE}_{tot} are the electrostatic, polarization, dispersive, repulsive, and total interaction energies, respectively.

Table S21: Interaction Energies for Selected Dimer Motifs Observed in **13** Crystal Structure[a]

motif	E^{CE}_{ele}	E^{CE}_{pol}	E^{CE}_{dis}	E^{CE}_{rep}	E^{CE}_{tot}
I	-22.8	-8.0	-52.5	28.9	-52.3
I''	-13.5	-6.3	-41.4	22.4	-37.1
II	-10.4	-8.7	-139.1	35.9	-112.4
VI	-20.8	-6.3	-92.0	44.2	-72.4
VII	-16.2	-7.7	-165.5	58.8	-123.1

[a] E^{CE} represents the interaction energies computed with CrystaExplorer. E^{CE}_{ele}, E^{CE}_{pol}, E^{CE}_{dis}, E^{CE}_{rep} and E^{CE}_{tot} are the electrostatic, polarization, dispersive, repulsive, and total interaction energies, respectively.

Table S22: Interaction Energies for Selected Dimer Motifs Observed in **VARGUR** Crystal Structure[a]

motif	E^{CE}_{ele}	E^{CE}_{pol}	E^{CE}_{dis}	E^{CE}_{rep}	E^{CE}_{tot}
I	-21.1	-8.3	-50.1	22.0	-54.1
I'	-20.7	-7.5	-49.5	16.0	-42.5
II	-15.2	-7.9	-149.6	50.4	-110.2
VII	-3.5	-1.3	-61.2	23.0	-40.8
IX	-3.8	-2.6	-48.7	16.7	-35.9

[a] E^{CE} represents the interaction energies computed with CrystaExplorer. E^{CE}_{ele}, E^{CE}_{pol}, E^{CE}_{dis}, E^{CE}_{rep} and E^{CE}_{tot} are the electrostatic, polarization, dispersive, repulsive, and total interaction energies, respectively.

Table S23: Interaction Energies for Selected Dimer Motifs Observed in **VARGOL** Crystal Structure[a]

motif	E_{ele}^{CE}	E_{pol}^{CE}	E_{dis}^{CE}	E_{rep}^{CE}	E_{tot}^{CE}
I	-20.7	-7.5	-49.5	16.0	-57.5
I'	-15.2	-8.1	-49.6	50.4	-55.2
II	-15.2	-8.1	-149.5	50.4	-115.2
III	-4.2	-5.4	-99.5	24.9	-77.2
VI	-4.3	-4.1	-61.6	10.1	-54.4
VII	-17.5	-7.8	-186.5	75.6	-129.6

[a] E^{CE} represents the interaction energies computed with CrystaExplorer. E_{ele}^{CE}, E_{pol}^{CE}, E_{dis}^{CE}, E_{rep}^{CE} and E_{tot}^{CE} are the electrostatic, polarization, dispersive, repulsive, and total interaction energies, respectively.

Table S24: Interaction energy between host-guest calculated by B3LYP-D3/6-31G** method in Gaussian16

**Guest**	**Eint [kJ/mol]**
Cyclohexane	-36.4
Benzen	-42.6
Toluene	-41.2
Cl2C=CCl2	-46.9
SCS	-46.0
PhCl	-38.2
PhBr	-37.0
Methyl acetate	-42.0
THF	-41.2
Dichloromethane	-53.3
Pyridine	-40.3
Acetonitrile	-32.3

Table S25: The **S** distance for TBC6 molecule in the **1** – **13** crystal structures

Structure	Solvent	CH2. . . CH2 Labels	**S**	σ(**S**)
**1**	benzene	C22 C55	4.884	0.003
**2**	pyridine 1:3	C22 C55	4.89	0.004
**3**	DCM 1:3	C22 C55	4.264	0.008
**4**	cyclohexane	C44 C11	4.878	0.005
**5**	toluene	C22 C55	4.883	0.009
**6**	methyl acetate	C11 C44	4.864	0.004
**7**	THF	C22 C55	4.823	0.004
**8**	pyridine 1:1	C11 C44	4.767	0.005
**9**	anisole	C22 C55	5.240	0.007
**10**	n-heptane	C22 C55	4.802	0.004
**11**	-	C22 C55	4.71	0.02
**12**	DCM 1:4	C22 C55	4.608	0.003
**13**	DCM 1:2	C33 C66	5.109	0.003
